# Benchmarking CO_2_ Adsorbents for Direct Air Capture with an Amine-Grafted Metal–Organic Framework

**DOI:** 10.1021/acscentsci.6c00769

**Published:** 2026-08-29

**Authors:** Jiaqi Zhang, Tianhao Chen, Aamir Hanif, Yuyan Shi, Xinyi Xu, Weixiang Zuo, Zhaolei Zhang, Yu Qiu, Paul Iacomi, Qiaowei Li, Yue-Biao Zhang

**Affiliations:** † Institute of Carbon Neutrality, School of Physical Science and Technology, Shanghai Key Laboratory of High-Resolution Electron Microscopy, State Key Laboratory of Advanced Medical Materials and Devices, 387433 ShanghaiTech University, Shanghai 201210, China; ‡ Department of Chemistry, State Key Laboratory of Porous Materials for Separation and Conversion, Shanghai Key Laboratory of Molecular Catalysis and Innovative Materials, Advanced Institute for Future Energy, 12478 Fudan University, Shanghai 200433, China; § Surface Measurement Systems, Unit 5, Wharfside, HA0 4PE, London, U.K.

## Abstract

Direct air capture (DAC) is a promising strategy for mitigating atmospheric CO_2_ accumulation, yet sorbent evaluation remains dominated by equilibrium uptake under static conditions that fail to capture kinetic, humid, and process-level constraints relevant to real operation. Here we establish a dynamic evaluation framework that replaces equilibrium-centric metrics with a unified platform integrating high-precision gravimetric measurements, ppm-level CO_2_ nondispersion infrared detection, and capacitive humidity sensing, enabling direct quantification of adsorption kinetics, H_2_O/CO_2_ coadsorption, breakthrough behavior, and regeneration energy under low CO_2_ concentration and dry/humid conditions. Using TAEA@MIL-101­(Cr) as a benchmark system, we introduce a sonication-assisted impregnation strategy that enables coordinated amine anchoring with preserved porosity and strong CO_2_ chemisorption. The optimized material exhibits a high equilibrium CO_2_ of 3.12 mmol g^–1^ (13.8 wt %) at 298 K, a dynamic breakthrough capacity of 12.4 wt % under dry conditions, and a retained capacity of 9.7 wt % at 50% relative humidity. Complete regeneration is achieved at 50 °C, enabling low-energy temperature-swing operation. This work establishes a generalizable dynamic benchmarking framework for DAC sorbents and positions TAEA@MIL-101­(Cr) as a reference platform bridging molecular design, kinetic performance, and process-level energy requirements to complement conventional equilibrium evaluation.

## Graphical abstract

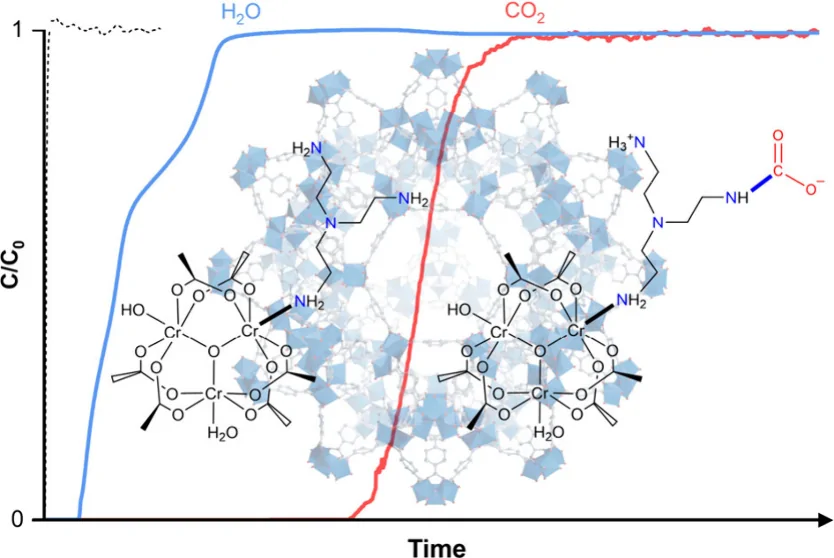

## Introduction

Direct air capture (DAC) is not only an indispensable technology for mitigating atmospheric CO_2_ but also a sustainable route for producing fuels within a circular carbon economy.^1–6^ Compared with conventional liquid absorption,^7,8^ solid adsorbents offer intrinsically lower energy penalties and greater process feasibility.^9–14^ However, the ultradilute concentration of atmospheric CO_2_ (∼420 ppm) combined with ubiquitous moisture imposes stringent design requirements on DAC adsorbents, including high low-pressure CO_2_ affinity, rapid mass transfer, and low regeneration energy.^15–22^

Metal–organic frameworks (MOFs) have emerged as a highly promising and tunable platform for DAC,^23–28^ owing to their controllable pore architectures and functionalizable pore environments,^29–36^ particularly when amine-functionalized for chemisorption under ambient conditions.^37–43^ Despite this potential, current evaluations of MOFs and other DAC adsorbents largely rely on static, single-component equilibrium measurements, which cannot fully reflect real-world kinetic uptake, diffusion limitations and dynamic breakthrough behavior under ultradilute humid continuous-flow conditions.^44,45^ As DAC progresses toward industrial deployment, adsorption kinetics and multicomponent competition (especially CO_2_/H_2_O) increasingly govern overall process efficiency, productivity, and energy consumption.^46–52^ Accordingly, rigorous characterization must prioritize coupled diffusion–chemisorption kinetics at ultradilute CO_2_ concentrations, competitive adsorption dynamics, regeneration energy, and cycling stability.^53–57^ MIL-101­(Cr) is chosen here as a mechanistic model rather than an industrial sorbent; despite poor scalability of Cr-based materials, its ordered pores and open metal sites support clear analysis of amine functionalization and CO_2_/H_2_O coadsorption.

Here, we establish a dynamic, flow-based evaluation platform that integrates breakthrough analysis with time-resolved gravimetric uptake profiling using a precision balance equipped with dedicated CO_2_ and humidity sensors.^58,59^ As widely pioneered by Brandani, Ruthven, and co-workers, standard volumetric or gravimetric uptake profiles measured in shallow beds often represent empirical, convolution-heavy responses governed by system-level nonisothermal heat dissipation, carrier gas flow rates, bed configurations, and localized competitive equilibria, rather than intrinsic molecular diffusion coefficients.^60,61^ Fully acknowledging these interpretive limits, our unified platform does not aim to extract idealized, intrinsic material constants via microscopic mathematical fitting. This platform enables simultaneous quantification of kinetic descriptors alongside equilibrium capacities under DAC-relevant conditions (ultralow CO_2_, carrier gases, and controlled humidity), providing a multidimensional and realistic assessment of adsorbent performance and directly informing practical applicability.

Guided by this protocol, we further develop a nanocomposite adsorbent, TAEA@MIL-101,^62–68^ via ultrasound-assisted impregnation of metal-coordinated polyamines within MOFs, achieving enhanced CO_2_ capture under continuous-flow conditions. TAEA@MIL-101­(Cr) demonstrates exceptional performance under practical DAC conditions, including a CO_2_ uptake of 3.12 mmol g^–1^ at 0.4 mbar and 298 K, a dynamic breakthrough capacity of 12.86 wt%, and regeneration at only 50 °C while retaining 92.2% CO_2_ capture over 10 cycles, substantially reducing thermal energy requirements. Notably, confinement of amine functionalities within the pore environment synergistically optimizes adsorption affinity and transport kinetics, enhancing dynamic capture efficiency while minimizing regeneration penalty.

This work not only advances the design of high-performance DAC adsorbents but, more importantly, establish a generalizable evaluation paradigm combining equilibrium characterization and dynamic kinetic testing to comprehensively assess practical adsorbent performance under realistic operating conditions, providing new insights into structure–performance relationships under realistic operating conditions.

## Experimental Section

### Synthesis of MIL-101­(Cr)

A mixture of Cr­(NO_3_)_3_·9H_2_O (3.0 g, 7.5 mmol) and H_2_BDC (1.26 g, 7.5 mmol) was prepared with 30 mL of deionized water and ultrasonicated to form a dispersion. The mixture was transferred to a Teflon-lined autoclave and heated to 200 °C for 16 h. The crude product was collected by centrifugation, washed with *N*,*N*-dimethylformamide (DMF) three times, and then immersed in DMF at 70 °C for 24 h to remove unreacted reagents. The obtained solid was washed with methanol (MeOH) three times to complete solvent exchange. Finally, the sample was activated under vacuum at 150 °C overnight.

### Synthesis and Activation of TAEA@MIL-101

100 mg of tris­(2-aminoethyl)­amine (TAEA) and 100 mg of activated MIL-101­(Cr) were separately dispersed in 2 mL of ethanol (EtOH) and sonicated for 5 min to achieve uniform dispersion. Under continuous sonication, the TAEA solution was added to the MIL-101­(Cr) dispersion, and sonication was continued for 30 min. Finally, the mixture was evaporated to dryness at 50 °C under a nitrogen atmosphere. The sample was activated overnight at 80 °C under vacuum. Elemental analysis: Calcd for [Cr_3_O­(OH)­(BDC)_3_(TAEA)_3.67_]·1.62TAEA·1.54H_2_O: C, 45.65; H, 7.65; N, 20.21%. Found: C, 45.20; H, 8.55; N, 20.37%. The sample was activated overnight at 80 °C under vacuum.

### Thermogravimetric Analysis (TGA)

TGA was conducted on a PerkinElmer TGA 4000 instrument at a heating rate of 10 °C min^–1^ from room temperature to 550 °C under nitrogen flow.

### Transmission Electron Microscopy and Energy Dispersive X-ray Spectroscopy (TEM-EDS)

TEM was conducted on a JEOL JEM-1400Plus transmission electron microscope equipped with EDS for mapping and line scan, to observe the morphology and elemental distribution of the samples.

### Adsorption Instrument

Gas adsorption experiments were conducted on a MicrotracBEL Belsorp Max2-SN88 high-throughput surface area analyzer and an Autosorb-iQ-MP-AG BET gas adsorption surface area analyzer.

### X-ray Photoelectron Spectroscopy (XPS)

XPS was conducted on a Thermo Scientific ESCALAB 250Xi X-ray photoelectron spectrometer to characterize the surface elemental composition and relative contents of the samples, and further analyze the coordination environment between MIL-101 and amine molecules. Before measurement, the samples were degassed under vacuum to remove residual solvents, and the powder samples were then pressed into pellets using a tablet press.

### Solid-State ^13^C HPDEC/MAS NMR

Solid-state ^13^C HPDEC/MAS NMR spectra were recorded on a Bruker AVANCE III HD 400 MHz spectrometer (9.4 T) using a 4.0 mm ZrO_2_ rotor at a spinning rate of 10 kHz. For ^13^CO_2_ dosing experiments, the activated sample was packed in an N_2_-filled glovebox, degassed under dynamic vacuum, and subsequently equilibrated with 1 bar of ^13^CO_2_ for 12 h. The chemical shifts were referenced to the carboxylic carbon of glycine at 176.2 ppm relative to TMS.

### Dynamic Vapor Sorption (DVS) Measurements

Dynamic CO_2_ uptake isotherms were gravimetrically recorded using a dynamic vapor sorption analyzer (DVS Carbon, Surface Measurement Systems) at 25 °C. Prior to measurements, ca. 10–20 mg samples were activated under high vacuum (<10^–5^ Torr) for 12 h, transferred to the DVS chamber, and further activated at 80 °C under a N_2_ flow (200 sccm) for 2 h. Upon cooling to 25 °C, the sample were equilibrated with a 400 ppm of CO_2_ flow (200 sccm) until the mass change rate (*dm*/*dt*) was less than 0.002% min^–1^.

### Heat of Adsorption by Experimental Measurement

The adsorption heat of CO_2_ was measured using Microtac BELsorp Max2 sorption analyzer coupled to a calorimetry tester (SenSys EVO). Heat changes were collected by thermal analysis. Differential scanning calorimeter (DSC) measurements were performed by heating and cooling the samples on a TA DSC Q2000 instrument, with a heat rate of 40 K min^–1^ under the N_2_ atmosphere.

### Breakthrough Measurements

Dynamic breakthrough experiments were conducted using a BTA Frontier (Surface Measurement Systems). Approximately 40 mg of the sample was packed into a silanized glass column (3 mm inner diameter) to form a fixed bed with a height of ca. 30 mm. Prior to the measurement, the sample underwent a strict activation protocol to ensure the complete removal of preadsorbed species: the material was first activated at 80 °C under high vacuum (10^–5^ Torr) for 20 h, followed by in situ activation at 80 °C under a pure N_2_ flow (50 sccm) for 2 h. After cooling to the adsorption temperature of 25 °C, the gas stream was switched from N_2_ to a feed gas containing 410 ppm of CO_2_ at a total flow rate of 50 sccm to initiate the adsorption process. The effluent gas concentration at the outlet was monitored using integrated NDIR sensor for CO_2_, a capacitive humidity sensor for H_2_O. To account for the sensor delay and the dead volume corrections blank adsorption and desorption experiments were carried out in same way except instead of the adsorbent a similar volume of glass beads were loaded in the column.

To evaluate the stability of the amine-functionalized adsorbent, the effluent gas during desorption was continuously monitored using an integrated Photoionization Detector (PID). PIDs are sensitive to amines and a broad range of volatile organic compounds (VOCs); however, given that the working temperature range following initial activation and subsequent adsorption is unlikely to volatilize organic species from our material other than amine-based degradation products, changes in PID signal can be predominantly attributed to amine leaching or decomposition. The PID signals were thus recorded to detect the elution of any volatile amine species or degradation products, serving as a semiquantitative indicator of material stability.

### CO_2_/H_2_O Competitive Adsorption Kinetics

To strictly evaluate and compare the adsorption rates, the apparent dynamic data were analyzed based on the intraparticle diffusion model. The slope of this curve serves as the kinetic parameter, and the square of the ratio of the slopes for the two guest molecules constitutes the apparent relative uptake rate ratio.^50^ The fractional uptake (*M_t_*/*M*_*e*_) during the initial adsorption stage is proportional to the square root of time, described by the following equation:

$$\frac{M_{t}}{M_{e}}=k\cdot t^{1/2}+C$$

Where:

*M* *_t_* is the adsorption uptake as the time-dependent;

*M_e_* is the adsorption uptake at equilibrium;

*k* is the intraparticle diffusion rate constant (slope), representing the diffusion rate;

C is a constant related to the boundary layer effect.

## Results and Discussion

### Optimized Loading Strategy and Structural Integrity of TAEA@MIL-101

Current evaluations of amine-functionalized adsorbents for direct air capture often overemphasize absolute amine loading and adsorption capacity, disregarding the detrimental pore clogging that hinders mass transfer kinetics. Inspiration for selecting the metal–organic framework MIL-101­(Cr),^62–68^ was based on the fact that its expansive mesoporous cages serve as an ideal host to harness spatial confinement, thereby balancing the inherent trade-offs between adsorption thermodynamics and mass transport kinetics. As illustrated in Scheme 1, the TAEA@MIL-101 composite was prepared through an ultrasound-assisted wet impregnation strategy. Through this optimized loading method, TAEA molecules are precisely confined within the mesoporous voids rather than aggregating on the exterior surface. This architecture maximizes the accessibility of amine active sites while preserving the structural integrity of the framework, which will be further elucidated in the subsequent sections. As shown in the SEM image (Figure 1a), the composite consists of regular octahedral particles resembling MIL-101­(Cr). Compared to the pristine porous support (Figures S1 and S2), the morphologies and sizes remained nearly intact after amine impregnation. Notably, no obvious particle agglomeration or amorphous surface coating was observed. To understand the amine distribution within the crystal, EDX line scans and maps were collected (Figure 1b and 1c), revealing that the N content remained nearly constant at different positions and exhibited uniform distributions of Cr and N, which confirms that amines were really loaded into MIL-101 pores and indicates the absence of aggregation.^69^ The structural integrity of the composite was further corroborated by powder X-ray diffraction (PXRD) and TGA (Figures S3 and S4). The TGA profiles indicate exceptional thermal stability up to approximately 280 °C, consistent with the robust Cr­(III)-carboxylate coordination network.

**Scheme 1:**
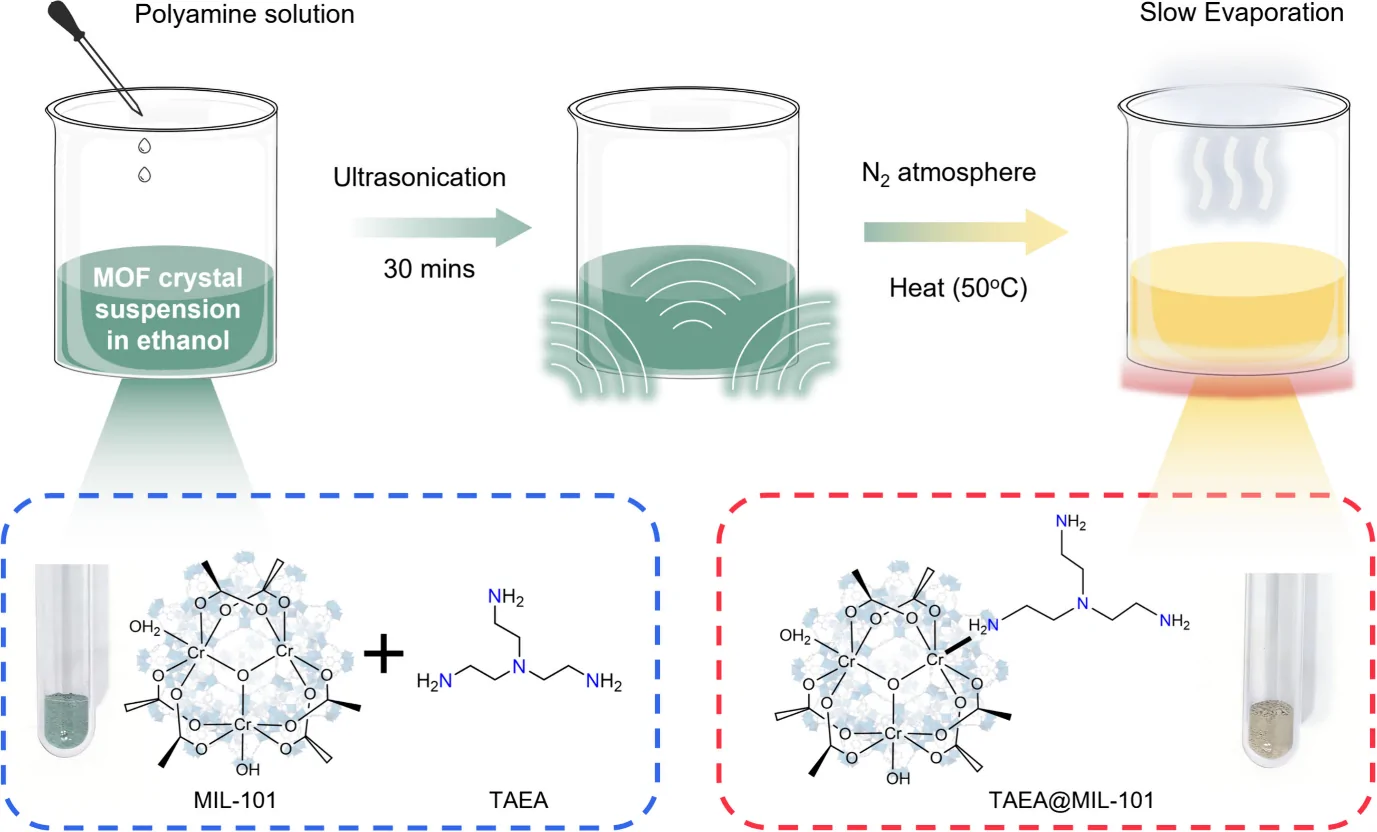
Ultrasound-Assisted Impregnation of Metal-Bound Polyamines for Optimized TAEA@MIL-101­(Cr) Nanocomposites in Direct Air Capture

**Figure 1:**
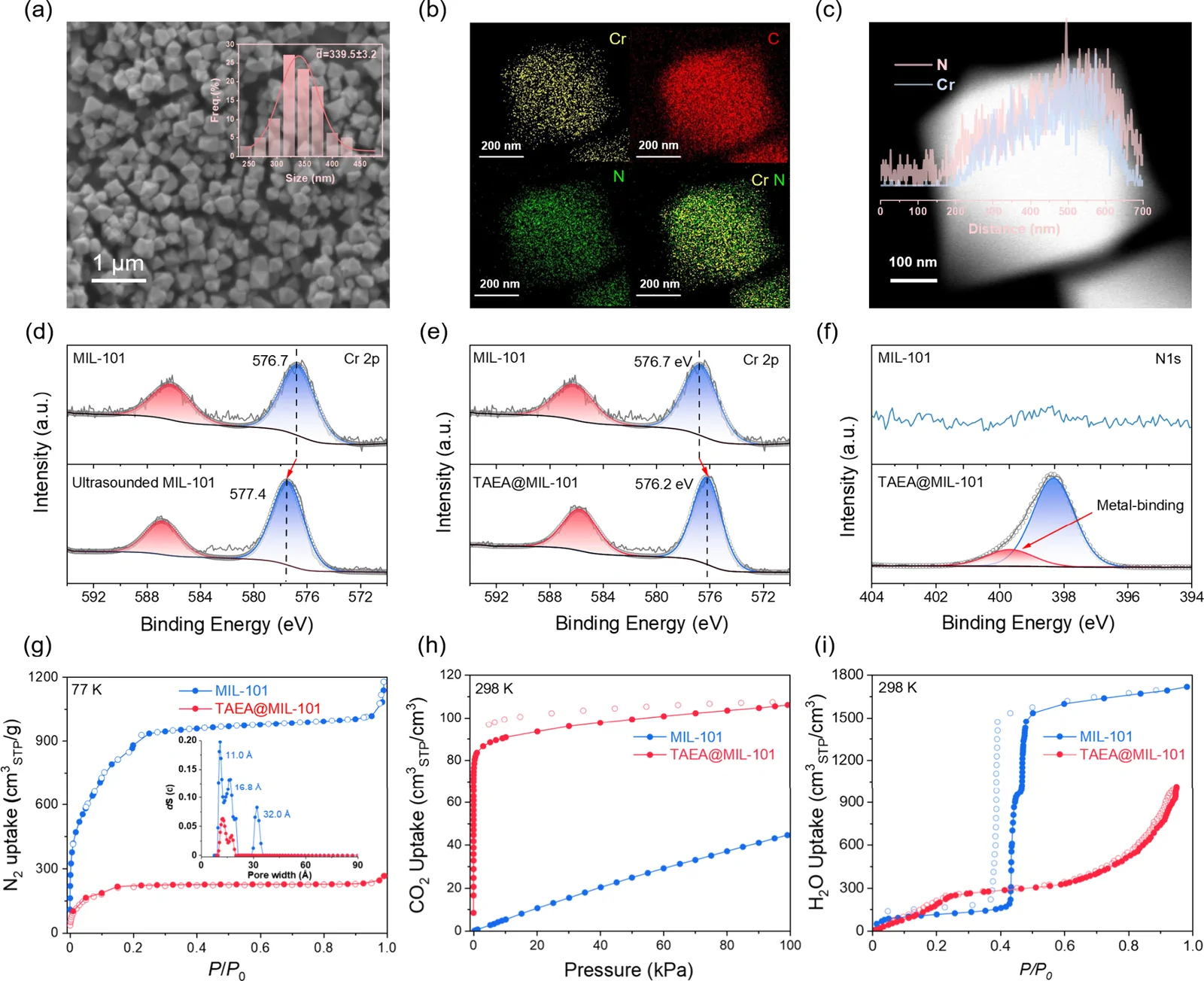
**Compositional, structural and porosity characterization of TAEA@MIL-101­(Cr), confirming optimal polyamine incorporation.** (a) SEM image showing uniform octahedral crystals after polyamine impregnation (inset: size distribution). (b) EDS elemental mapping (Cr, C, N), showing homogeneous polyamine distribution. (c) HAADF-STEM image and cross-sectional EDS line scan, confirming absence of surface blockage. (d) High-resolution XPS (Cr 2p) before and after sonication, indicating increased exposure of metal sites. (e) High-resolution XPS (Cr 2p) after polyamine impregnation, indicating coordination to amine species. (f) High-resolution XPS (N 1s), confirming incorporation of metal-bound amines. (g) N_2_ adsorption isotherms (77 K) with pore size distribution (inset), showing pore occupation relative to pristine MIL-101. (h) CO_2_ adsorption isotherms (298 K), showing enhanced uptake at low pressure relative to pristine MIL-101. (i) H_2_O adsorption isotherms (298 K), showing reduced hygroscopicity after amine incorporation.

Furthermore, the host–guest interaction was probed by XPS. N 1s spectrum displays characteristic amine peaks (Figure 1f) while the Cr 2p binding energy exhibits a distinct negative shift of ∼ 0.5 eV (from 576.7 to 576.2 eV, Figure 1e). This phenomenon is attributed to the electron donation from TAEA amine groups to the unsaturated Cr centers, forming robust coordinate bonds (N→Cr) to stabilize the guests.^70,71^ Notably, this efficient coordination is facilitated by the activation process, which effectively strips the precoordinated water molecules to expose electron-deficient open metal sites (as indicated by the intermediate positive shift to 577.4 eV, Figure 1d), thereby driving the subsequent amine substitution.

Upon amine loading, the specific surface area decreased significantly from 3300 to 840 m^2^ g^–1^ (Table S1). The complete disappearance of characteristic mesoporous peaks in the pore size distribution (PSD) profiles, accompanied by the partial retention of micropores, unambiguously confirms that TAEA molecules are deeply confined within the mesoporous voids (Figure 1g). Despite this reduction in textural porosity, the introduction of abundant amine active sites drastically reverses the adsorption behavior. In stark contrast to the linear physisorption profile of pristine MIL-101, the steep Type-I isotherm of the composite at low pressures highlights the efficacy of the spatially confined amines. Consequently, TAEA@MIL-101 exhibits a steep CO_2_ uptake at extremely low partial pressures (Figure 1h), unlocking the critical capability to capture trace-level CO_2_. Concurrently, the water vapor uptake is significantly suppressed (Figure 1i), indicating an enhanced hydrophobicity. This synergistic combination of ultralow-pressure CO_2_ affinity and moisture resistance establishes a robust foundation for practical direct air capture.

The strategic selection of TAEA as the amine modifier rather than conventional polymeric polyethylenimine (PEI) or linear polyamines (e.g., TEPA) stems from its optimal molecular balance between chemical capture affinity and mass transport kinetics. Polymeric components like PEI, though nonvolatile, often suffer from molecular entanglement and random aggregation, blocking the mesoporous entry windows of the host framework and deteriorating the overall amine efficiency. In contrast, the tripodal branched geometry of TAEA prevents tight intramolecular packing under spatial confinement, guaranteeing that its high density of reactive terminal primary amine sites remains fully accessible to diffuse trace CO_2_ molecules. Furthermore, the strong electron-donating nature of its nitrogen atoms facilitates multidentate coordination with the open Cr­(III) metal nodes.

### Exceptional CO_2_ Uptake Capacity and Chemisorption Mechanism

The thermodynamic behavior of CO_2_ capture for TAEA@MIL-101 was evaluated from static adsorption isotherms at varying temperatures. The material exhibited a remarkably high adsorption affinity and an exceptional uptake capacity of 3.12 mmol g^–1^ at an ultralow partial pressure of 0.04 kPa (400 ppm) at 298 K, demonstrating its robust potential across a broad temperature window. (Figure 2a). The dynamic adsorption performance was subsequently demonstrated via DVS measurements. Under a continuous flow of 400 ppm of CO_2_ at 298 K, the material achieves a high dynamic capacity of 12.8 wt % and reaches equilibrium rapidly in less than 100 min. (Figure 2b and Figure S7). Notably, efficient CO_2_ adsorption can be achieved at different CO_2_ concentrations (Figure S8), corroborating its practical viability for direct air capture.

**Figure 2:**
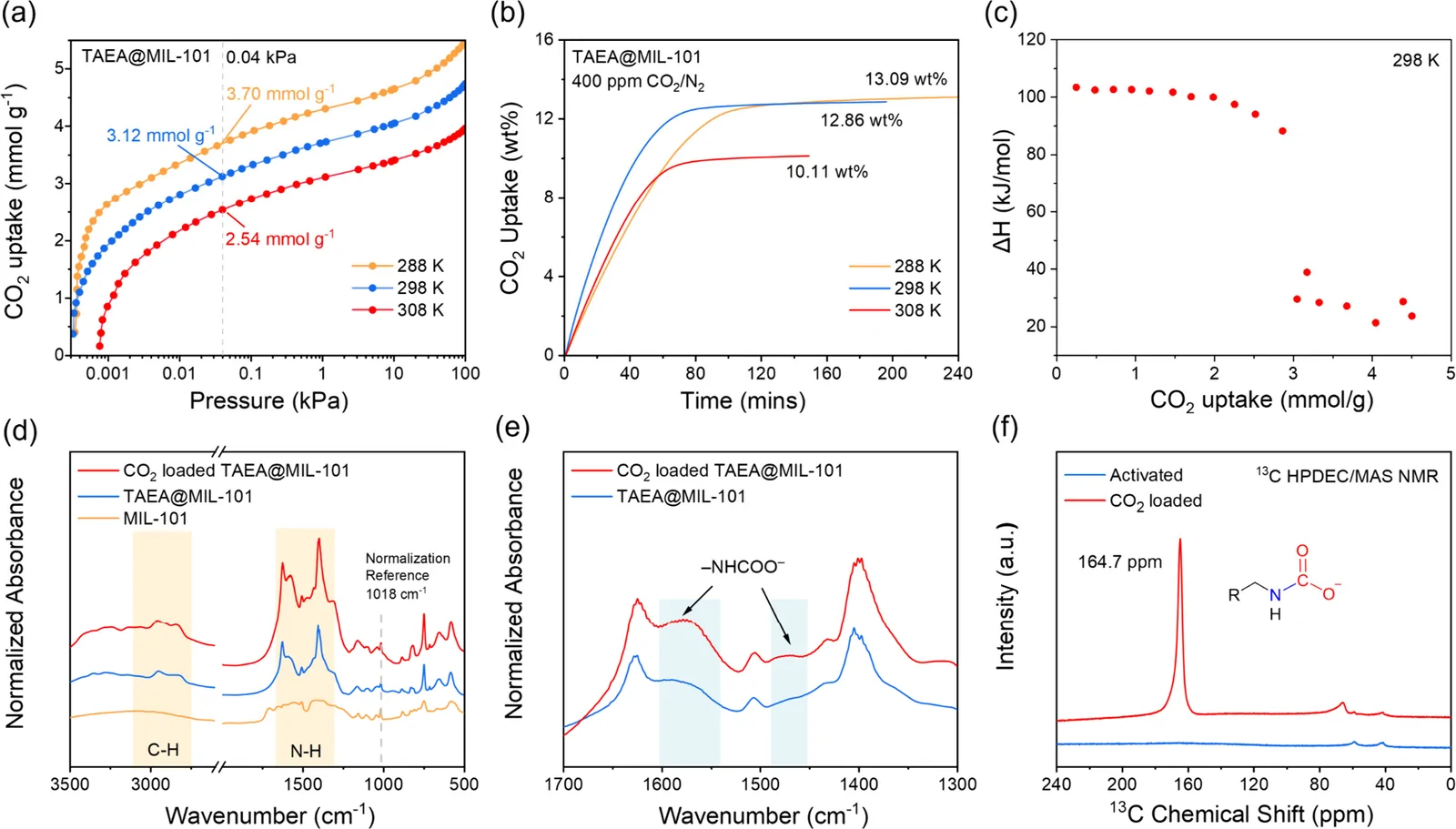
**CO**_**2**_ **capture performance and mechanistic investigation of TAEA@MIL-101­(Cr), establishing chemical adsorption for direct air capture.** (a) CO_2_ adsorption isotherms (at 288, 298, and 308 K), showing steep uptake at ultralow pressures relevant to DAC. (b) CO_2_ uptake at 400 ppm (at 288, 298, and 308 K), showing rapid adsorption kinetics. (c) *In situ* microcalorimetry at 298 K, revealing high adsorption enthalpy characteristic of chemical adsorption. (d) FT-IR spectra of MIL-101, TAEA@MIL-101, and CO_2_-loaded TAEA@MIL-101, confirming amine incorporation (normalized to 1018 cm^–1^). (e) FT-IR (1300–1700 cm^–1^), showing formation of −NH–COO^–^ species upon CO_2_ chemisorption. (f) Solid-state ^13^C NMR (HPDEC/MAS) before and after ^13^CO_2_ loading, confirming carbamate formation.

The host–guest interaction strength was quantified by direct calorimetric measurements of the adsorption heat, with the resulting profiles corroborating the chemisorption mechanism (Figure 2c). To gain molecular-level insights into the chemisorption mechanism suggested by the thermodynamic data, Fourier-transform infrared (FT-IR) spectroscopy was performed. For rigorous comparability, the spectra were normalized against the robust framework vibration band at 1018 cm^–1^ as an internal reference (Figure 2d). As illustrated in the magnified region (Figure 2e and Figure S5), pristine TAEA@MIL-101 exhibits characteristic δ NH_2_ and ν N–H vibrations at 1595 cm^–1^ and 3200–3400 cm^–1^, respectively, confirming the successful loading of the TAEA molecules. In distinct contrast, the CO_2_-dosed sample displays prominent new bands in this region. These diagnostic peaks are unambiguously assigned to carbamate species (−NH–COO−).^72,73^

This chemisorption mechanism was further corroborated by solid-state ^13^C NMR (Figure 2f), verifying the specific bonding between CO_2_ and TAEA.^74^ Following exposure to ^13^C-labeled CO_2_, a sharp and narrow peak at 164.7 ppm. This signal serves as a characteristic fingerprint of the carbonyl carbon within ammonium carbamate species. The direct NMR evidence, in conjunction with the FT-IR anaysis, unequivocally confirms that the TAEA@MIL-101 captures CO_2_ via a chemical pathway, culminating in the formation of stable ammonium carbamate ion pairs.

### Adsorption Kinetics, Uptake Capacity, and CO_2_/H_2_O Selectivity under Humid Conditions

Real-world direct air capture deployment inevitably faces the challenge of moisture. To evaluate the CO_2_/H_2_O separation performance under practical humid conditions, time-dependent CO_2_ and H_2_O uptake profiles were recorded at varying relative humidities (Figure 3a). As depicted in the raw uptake curves, the two adsorbates exhibit distinct kinetic behaviors upon initial exposure. To qualitatively compare relative adsorption trends among samples under fully unified experimental conditions, uptake curves were fitted via the intraparticle diffusion model. The obtained slope is only an apparent empirical parameter for internal comparison within this work under identical flow, temperature and sample loading; such fitted values cannot be treated as intrinsic material diffusion descriptors.^75,76^ The measured initial uptake slope of H_2_O is steeper than dilute CO_2_ under identical humid flow conditions, reflecting integrated overall mass transfer signals rather than intrinsic molecular diffusion rates. Quantitative comparison of intraparticle diffusion constants at 30%, 50% and 80% RH (Tables S2–S4) shows the H_2_O/CO_2_ kinetic selectivity reaches 7.17 at 50% RH and further rises to 8.65 at 80% RH, quantitatively confirming much faster mass transfer of water vapor during coadsorption. These numerical discrepancies directly illustrate how humidity modulates dynamic CO_2_ working capacity, strongly demonstrating that adsorption kinetics, instead of equilibrium uptake, dominate practical DAC performance under dilute humid air.

**Figure 3:**
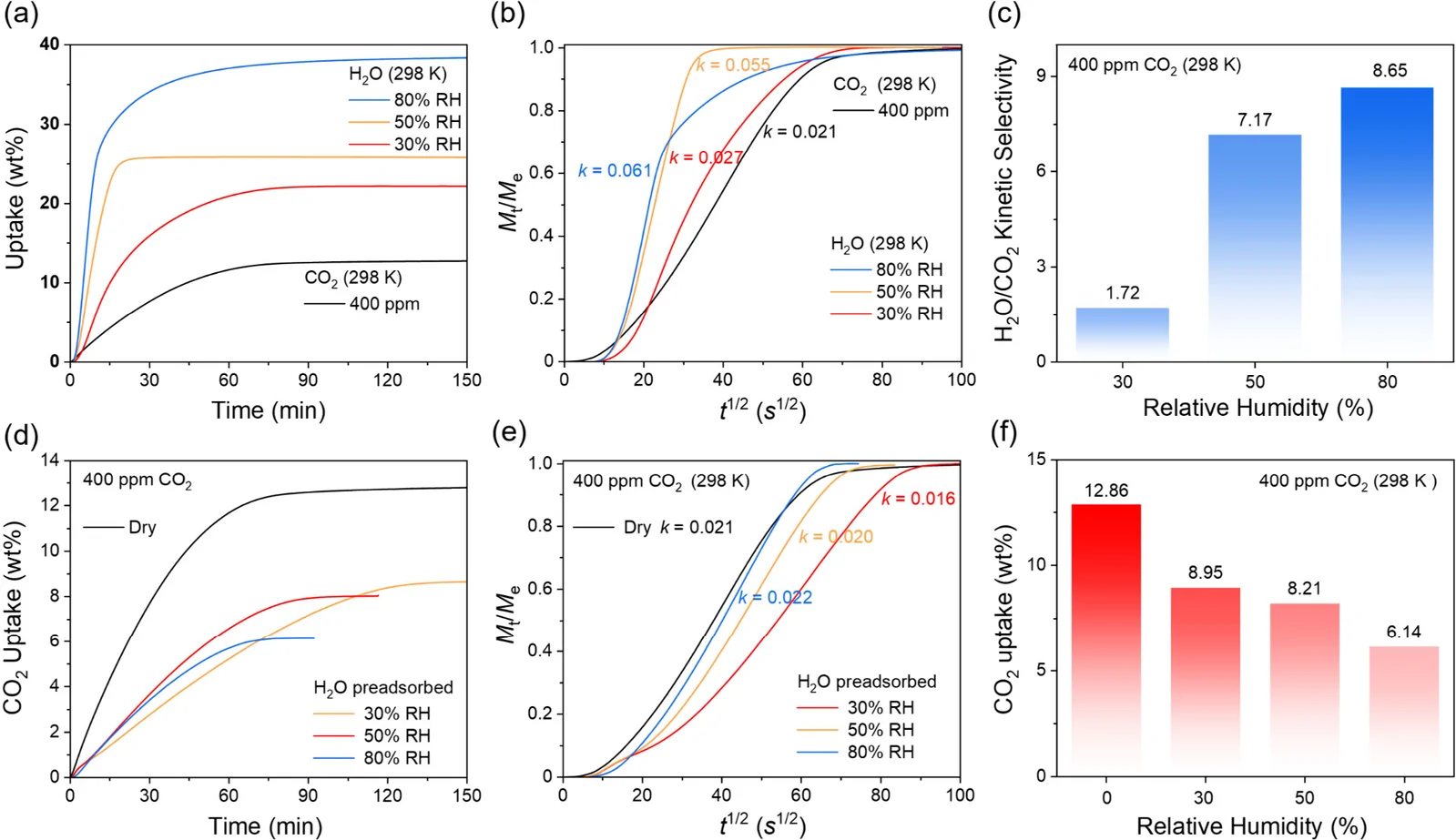
**H**_**2**_ **O/CO**_**2**_ **competitive adsorption and kinetic selectivity of TAEA@MIL-101­(Cr) under humid conditions approaching direct air capture.** (a) H_2_O uptake at 30%, 50%, and 80% RH vs 400 ppm of CO_2_, showing faster H_2_O adsorption kinetics. (b) Intraparticle diffusion fitting, yielding apparent diffusion rate constants (*k*). (c) H_2_O/CO_2_ kinetic selectivity as a function of relative humidity, quantifying competitive adsorption. (d) CO_2_ uptake (400 ppm) after H_2_O preadsorption (30–80% RH), retaining high capacity under humid conditions. (e) Intraparticle diffusion analysis of CO_2_ uptake, showing humidity-dependent CO_2_ adsorption kinetics. (f) Equilibrium CO_2_ uptake under dry and humid conditions (30–80% RH), retaining substantial capacity.

Following the kinetic evaluation methodology detailed in the Supporting Information, these dynamic profiles were further analyzed. To deconvolute the competitive H_2_O/CO_2_ adsorption dynamics. In Figure 3a, a comparative analysis based on the intraparticle diffusion model reveals substantial disparities in the diffusion rates of the two species. The fitted slopes represent comprehensive apparent response signals affected by competitive adsorption, thermal gradients and bed mass transfer. Kinetic analysis using the intraparticle diffusion model (Figures 3b) reveals that due to the combined influence of multiple factors, including competitive adsorption, amine–water hydrogen bonding, molecular size and polarity, exhibit significantly faster apparent diffusion kinetics (*k*_H_2_O_) than dilute CO_2_ (*k*_CO_2__) across the humidity range of 30–80% RH (Table S2). For instance, at 50% RH, *k*_H_2_O_ surpasses *k*_CO_2__ by over 2-fold, implying that moisture rapidly occupies the mesoporous channels prior to significant CO_2_ uptake.^50^

Sequential adsorption experiments confirmed that the accessibility of the amine sites remains unimpaired by preadsorbed water, ensuring the structural availability of active sites even under moisture-saturated conditions. Even after the material was pre-equilibrated with water vapor (at 30%, 50% and 80% RH), the subsequent introduction of 400 ppm of CO_2_ triggered a rapid and substantial secondary mass gain (Figure 3d). The intraparticle diffusion fitting for this secondary CO_2_ uptake phase (Figure 3e and Table S3) confirms that the internal amine sites remain highly accessible to CO_2_ molecules. The ultimate impact of moisture on the mixed-gas adsorption capacity is rigorously quantified (Figure 3f). Notably, TAEA@MIL-101 demonstrates exceptional humidity tolerance, retaining a high CO_2_ working uptake capacity of 8.95 wt % under a 400 ppm of CO_2_ stream even after reaching saturation with moisture at 50% RH (Figure 3d and Table S3). Furthermore, comparative kinetic analysis reveals that the extensive coadsorption of water does not severely inhibit the subsequent CO_2_ uptake (Tables S4). This close similarity in kinetic parameters (*k*_H_2_O_) suggests minimal competitive interference between H_2_O and CO_2_ molecules within the amine-functionalized mesoporous channels (Figure 3f). This behavior indicates that the amine-mediated chemisorption process remains highly effective regardless of the CO_2_/H_2_O adsorption order, ensuring reliable carbon capture from humid gas streams.^46–52^ This relative independence between the two species facilitates a robust carbon capture performance, even under the competitive constraints of realistic humid DAC conditions.

### Practical Viability in Direct Air Capture and Comprehensive Benchmarking

The practical applicability of TAEA@MIL-101 was evaluated via dynamic gas-adsorption breakthrough experiments using simulated ambient air (400 ppm of CO_2_ balanced with N_2_, Figure 4a, b). Specifically, packed beds containing 40 mg and 39 mg of the adsorbent were tested under dry and humid (50% RH) conditions, respectively.

**Figure 4:**
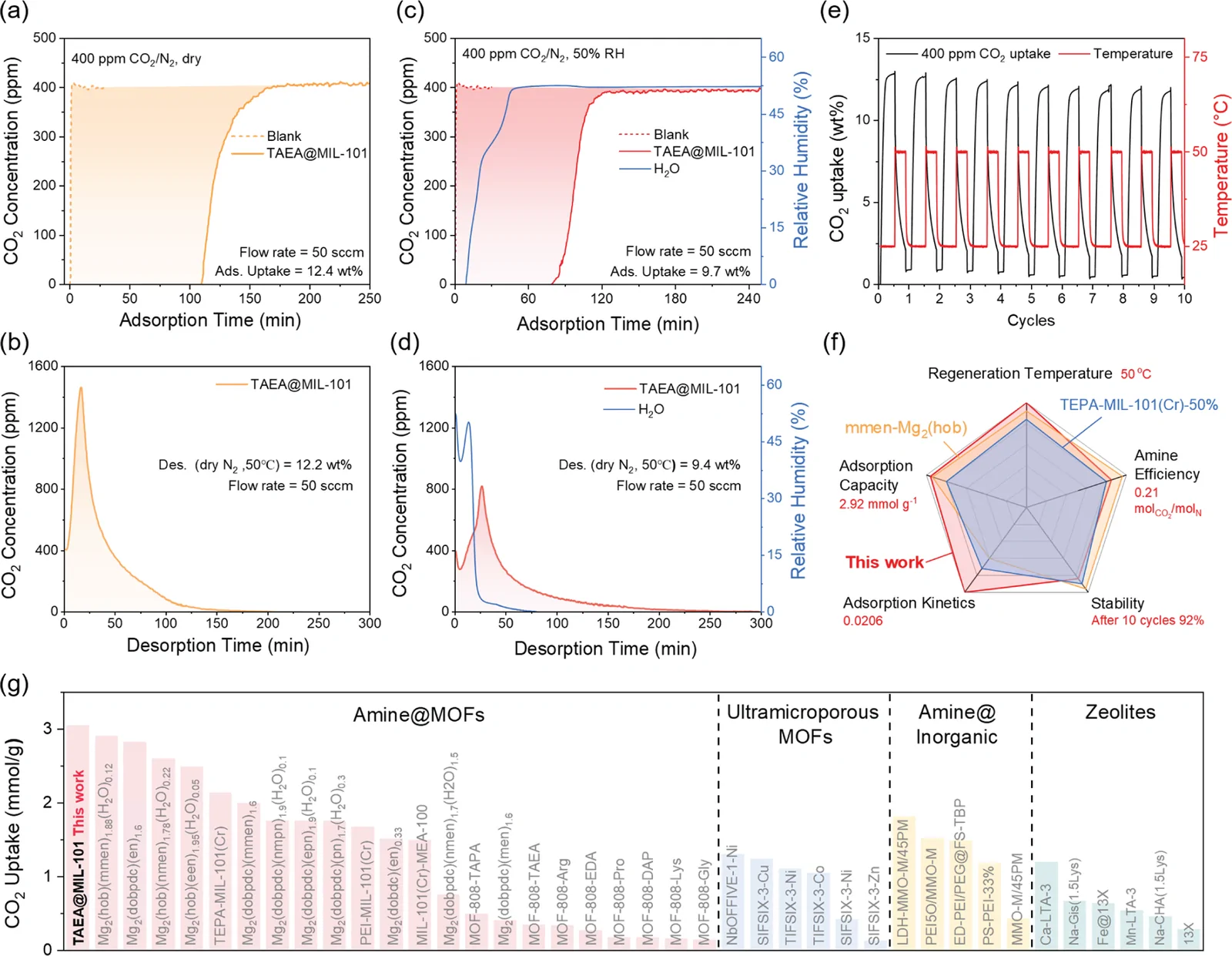
**Practical direct air capture performance of TAEA@MIL-101­(Cr): breakthrough behavior, regeneration, cyclic stability, and benchmarking.** (a) CO_2_ breakthrough (400 ppm of CO_2_/N_2_, dry), showing fast kinetics and extended CO_2_ retention (12.4 wt %). (b) CO_2_ desorption under, achieving complete regeneration at 50 °C. (c) CO_2_/H_2_O breakthrough (400 ppm of CO_2_/N_2_, 50% RH), retaining high CO_2_ capacity (9.7 wt %) under humid conditions. (d) CO_2_ and H_2_O desorption profiles, confirming full regeneration at 50 °C. (e) Temperature-swing adsorption (25–50 °C) over 10 cycles, showing stable performance and low-temperature regeneration. (f) Radar comparison of DAC metrics (capacity, kinetics, stability, amine efficiency, regeneration energy), highlighting balanced performance. (g) Benchmarking of CO_2_ uptake (400 ppm, 298 K) against state-of-the-art adsorbents, establishing TAEA@MIL-101­(Cr) as a leading platform for direct air capture.

Strikingly, TAEA@MIL-101 exhibits sharp breakthrough fronts, with the breakthrough time under humid conditions remaining nearly identical to that observed under dry conditions. A slight rollup of water does indicate slight water displacement in the presence of CO_2_. This kinetic consistency is of paramount significance within our DAC evaluation framework: the material maintains a substantial dynamic capacity of 9.69 wt % at 50% RH even with a preadsorbed moisture content of 34.96 wt%—retaining the vast majority of its dry capacity (12.39 wt %). This result rigorously confirms the high degree of site-specific immunity to moisture interference, representing a critical technical advantage for practical deployment.

Reducing regeneration energy is a key metric for minimizing the operating expenditure associated with DAC systems. The regeneration process of TAEA@MIL-101 was implemented by heating the column to 50 °C under N_2_ purge (Figure 4c, d). As revealed by the desorption profiles, the enriched concentration CO_2_ was released rapidly, and complete desorption was achieved within approximately 100 min of real time.

This ultralow regeneration temperature is of paramount value within our evaluation framework: it not only significantly mitigates the parasitic energy penalty but also enables direct thermal integration with low-grade industrial waste heat. This stands in stark contrast to conventional amine-based adsorbents, which typically necessitate much high regeneration temperatures.

Notably, real-time monitoring of the effluent gas via a photoionization detector (PID) during the regeneration phase reveals negligible signals for volatile amine species, thereby corroborating the robust spatial confinement of TAEA and ruling out significant amine leaching (Figure S12).

The long-term durability of an adsorbent serves as the ultimate benchmark for assessing its potential for practical deployment.^53–57^ TAEA@MIL-101 demonstrated exceptional stability with negligible capacity attenuation over 10 consecutive adsorption–desorption cycles, confirming its robustness for sustained DAC operations (Figure 4e and Figure S9). Comparative benchmarking against an extensive library of representative adsorbents (Figure 4g and Table S5) reveals the superior CO_2_ uptake of TAEA@MIL-101, positioning it among the top-tier performers across diverse material categories. However, practical viability necessitates more than just high capacity. As illustrated in the radar plot (Figure 4f), TAEA@MIL-101 exhibits a balanced and exceptional profile across all critical performance metrics.

## Conclusion

In summary, this work establishes a strategic blueprint for the rational design and industrial feasibility assessment of high-performance direct air capture adsorbents by leveraging the spatial confinement of long-chain polyamines within mesoporous frameworks. The encapsulation of TAEA into the MIL-101­(Cr) cages yields a superior dynamic CO_2_ breakthrough capacity of 12.39 wt% under simulated ambient air (400 ppm), demonstrating that site-specific structural optimization can effectively circumvent the pore-clogging issues and kinetic limitations that typically plague traditional amine impregnation methods.

Beyond CO_2_ uptake capture, we have introduced a comprehensive evaluation protocol to rigorously vet the industrial potential of TAEA@MIL-101. This systematic assessment confirms that TAEA@MIL-101 achieves a rare equilibrium among high dynamic capture efficiency, robust moisture immunity, and ultralow-temperature regeneration. By excelling across these diverse metrics, this study not only presents a top-tier candidate for large-scale carbon management but also establishes a new performance benchmark and a standard evaluation framework for the next generation of direct air capture technologies.

Such insight directly guides the rigorous optimization of low-temperature swing trajectories that minimize high-grade heat demand while mitigating thermal-driven amine degradation. More importantly, the concurrent monitoring of coadsorbed water vapor desorption offers a strategic avenue to directly evaluate and deconvolute the parasitic sensible and latent heat penalties that dominate the energetic viability of humid direct air capture systems, bridging the gap between fundamental chemistry and practical engineering design.

## Supplementary Material



## Notes

Synthetic details, static gas/vapor adsorption isotherms, CO_2_/H_2_O competitive adsorption kinetics, mimic direct air capture environment adsorption breakthrough, TGA, PXRD, SEM, XPS, and *Q*_st_ (PDF)
