# Supplementary material for: Benchmarking CO2 Adsorbents for Direct Air Capture with an Amine-Grafted Metal–Organic Framework
Source: ACS Cent Sci. 2026 Aug 29;12(9):1418–28. doi: 10.1021/acscentsci.6c00769 (PMC13614033; doi:10.1021/acscentsci.6c00769)
Supplement: Supplementary file 1 [file oc6c00769_si_001.pdf]

Supporting Information（27 pages）

Benchmarking CO_2 Adsorbents for Direct Air Capture with an Amine-Grafted Metal–Organic Framework

Jiaqi Zhang,[†] Tianhao Chen,[‡] Aamir Hanif,[§] Yuyan Shi,[†] Xinyi Xu,[†] Weixiang Zuo,[†] Zhaolei Zhang,[†] Yu Qiu,[†] Paul Iacomi,[*,§] Qiaowei Li,[*,‡] and Yue-Biao Zhang[*,†]

[†]*Institute of Carbon Neutrality, School of Physical Science and Technology, Shanghai Key Laboratory of High-Resolution Electron Microscopy, State Key Laboratory of Advanced Medical Materials and Devices, ShanghaiTech University, Shanghai 201210, China.*

[‡]*Department of Chemistry, State Key Laboratory of Porous Materials for Separation and Conversion, Shanghai Key Laboratory of Molecular Catalysis and Innovative Materials, Advanced Institute for Future Energy, Fudan University, Shanghai 200433, China.*

[§]*Surface Measurement Systems, Unit 5, Wharfside, HA0 4PE, London, UK.*

**To whom correspondence should be addressed. E-mail: zhangyb@shanghaitech.edu.cn, qwli@fudan.edu.cn, or PIaco-mi@surfacemeasurementsystems.com*

Table of Contents

Section S1. Synthesis and Characterization 3

Section S2. Dynamic Vapor Sorption (DVS) Measurements 11

Section S3. CO_2/H_2O Competitive Adsorption Kinetics 15

Section S4. Mimic Direct Air Capture Environment Adsorption Breakthrough Measurements 21

Section S5. Comparative Analysis of CO_2 Capture Performance 22

References 26

Section S1. Synthesis and Characterization

Materials: Chromium(III) nitrate nonahydrate [$Cr(NO_3)_3 \cdot 9H_2O$] (purity 99%) and terephthalic acid [H_2BDC] (purity 99%) were purchased from Aladdin Bio-Chem Tech. Co.; Tris(2-aminoethyl)amine [TAEA] (purity 98%) was purchased from Leyan; N,N-Dimethylformamide [DMF] (purity 99%) was purchased from Greagent; Anhydrous methanol [MeOH] (analytical reagent, purity ≥ 99.5%) and anhydrous ethanol [EtOH] (analytical reagent, purity ≥ 99.5%) were purchased from Sinopharm Chemical Reagent Co. All the starting materials mentioned above were used as received without further purification.

Characterization and Instrumentation.

The phase purity and crystallinity of samples was determined by a powder X-ray diffractometer (Bruker, D8 advance, Cu Kα). The crystal size and morphology were examined using a scanning electron microscope (JEOL, JSM 7800F Prime). Thermogravimetric analyses (TGA) were performed on a TGA instrument (Perkin-Elmer, TGA 4000) with a heating rate of 10 °C·min^{-1} from room temperature to 550 °C under N_2 flow. Infrared spectra were collected on FT-IR Spectrometer (Perkin-Elmer, Frontier). Solid-state ^{13}C HPDEC/MAS NMR spectra were recorded on a Bruker AVANCE III HD 400 MHz spectrometer (9.4 T) using a 4.0 mm ZrO_2 rotor at a spinning rate of 10 kHz. For $^{13}CO_2$ dosing experiments, the activated sample was packed in an N_2-filled glovebox, degassed under dynamic vacuum, and subsequently equilibrated with 1 bar of $^{13}CO_2$ for 12 h. The chemical shifts were referenced to the carboxylic carbon of glycine at 176.2 ppm relative to TMS.

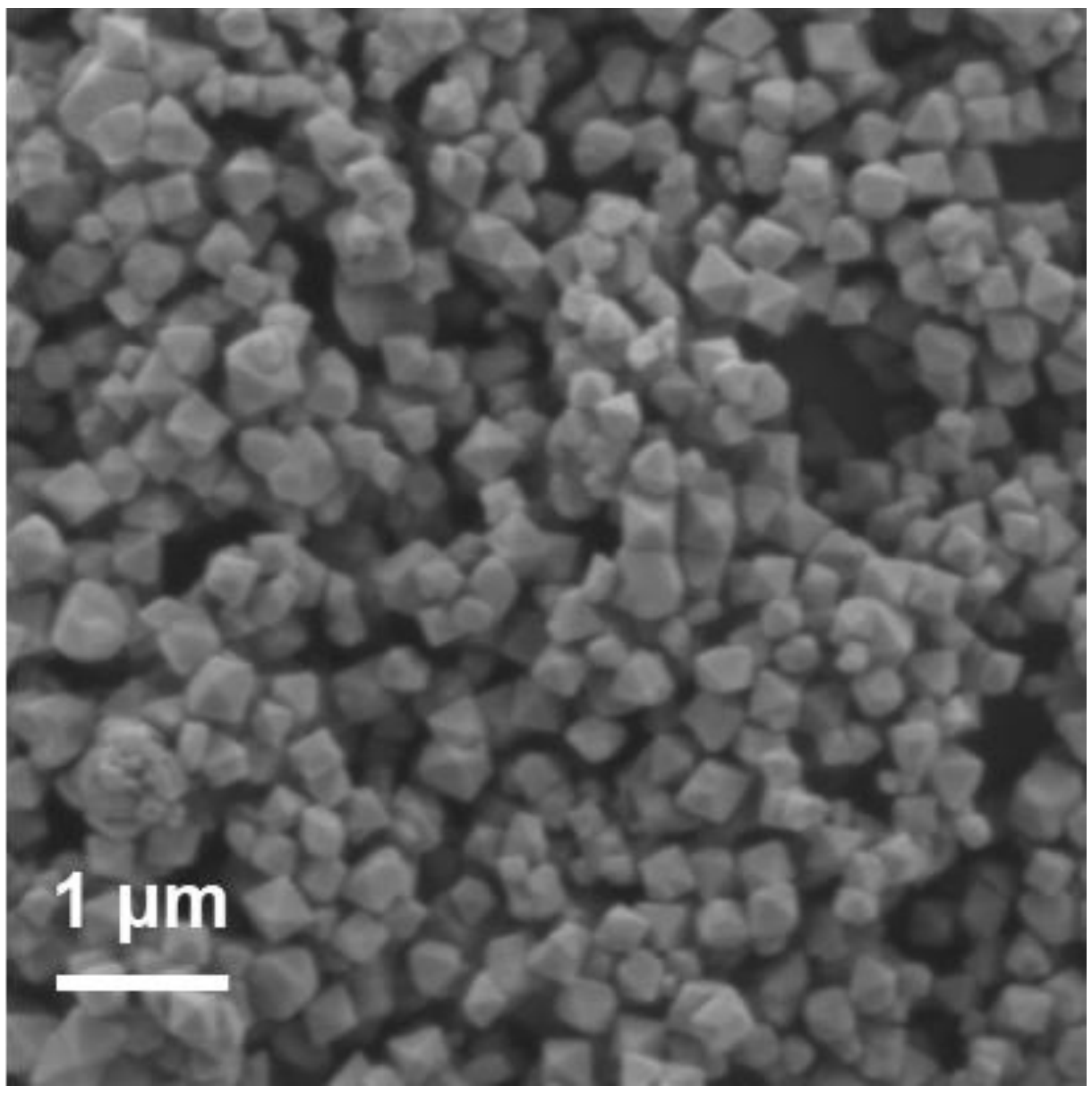

Figure S1. SEM image of MIL-101 shows well-defined octahedral morphology.

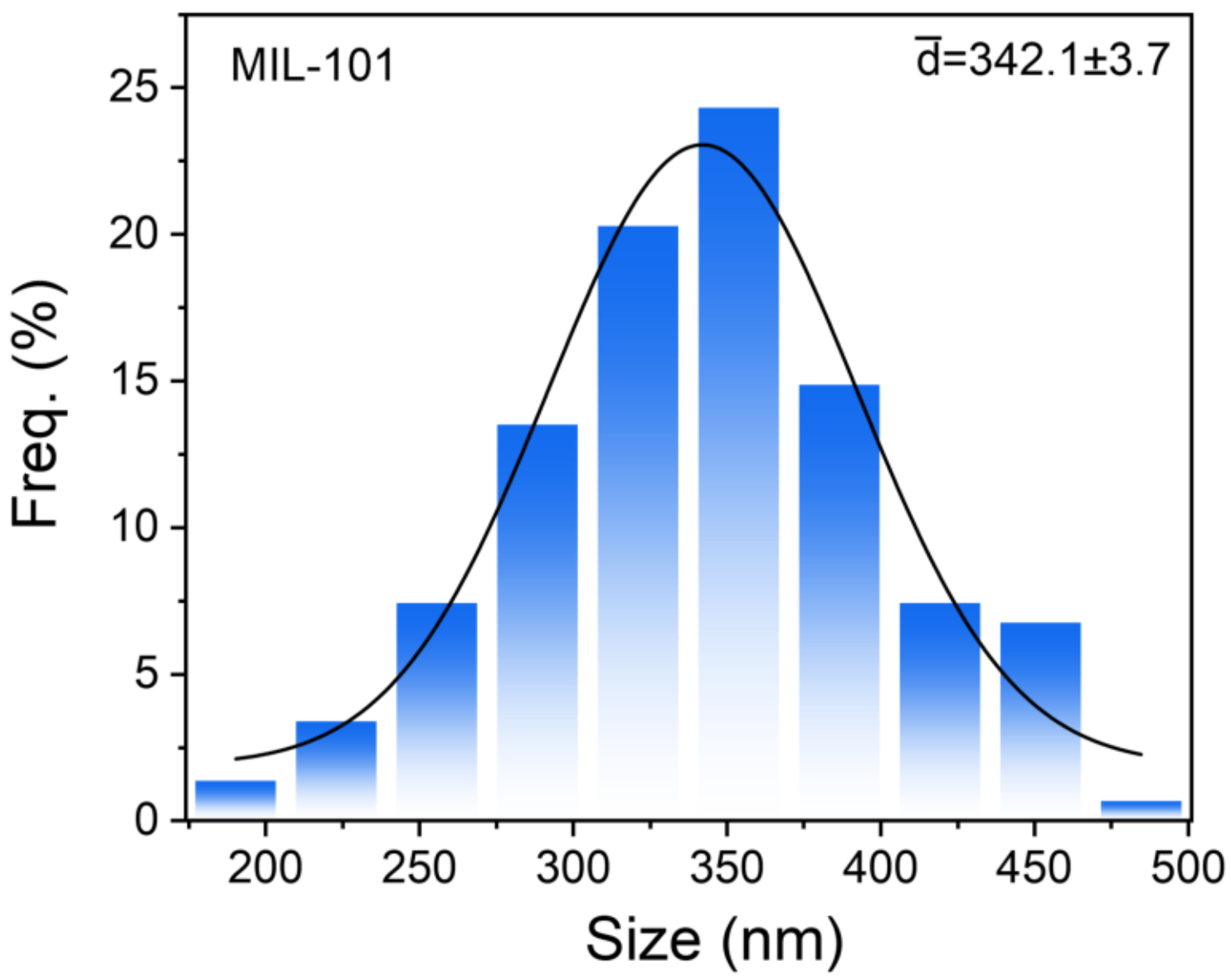

Figure S2. Size distribution of MIL-101.

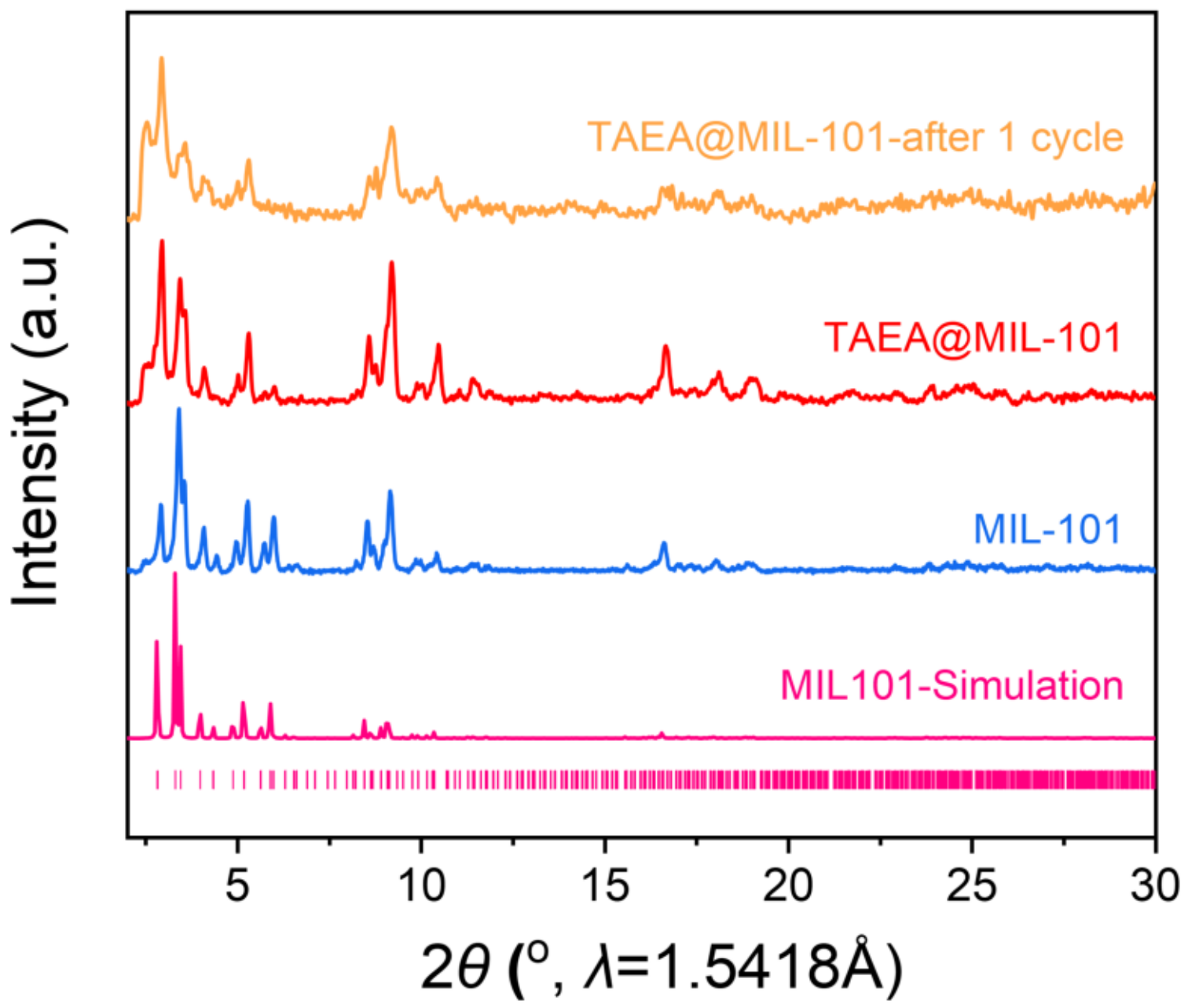

Figure S3. PXRD patterns of MIL-101 and TAEA@ MIL-101.

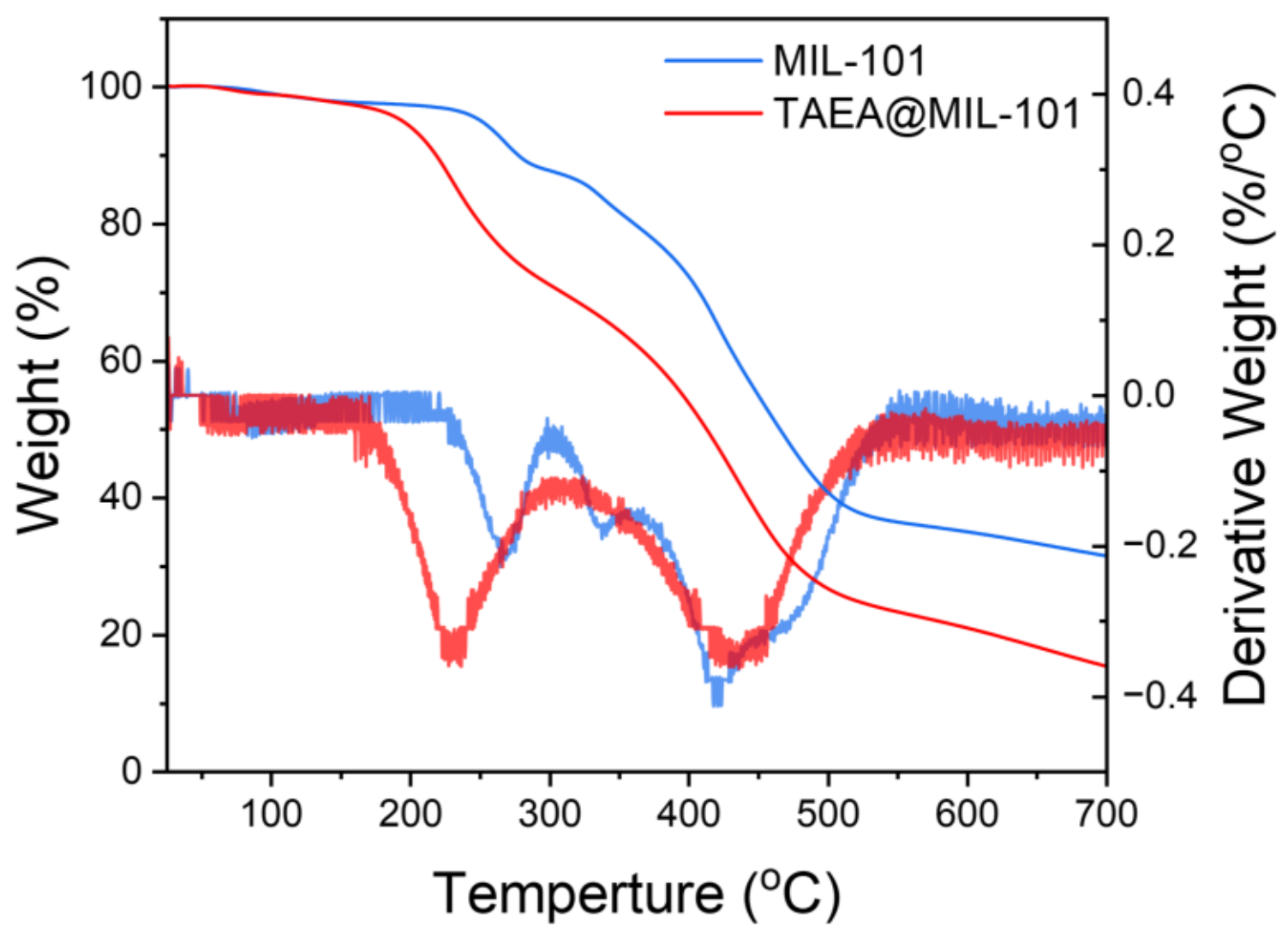

Figure S4. TGA curves of MIL-101 and TAEA@ MIL-101 under N_2 atmosphere.

Table S1. Porous properties of materials.

Sample	Total pore volume (cm^3/g)	BET surface area (m^2/g)
**MIL-101**	1.61	3300
**TAEA@MIL-101**	0.41	840

To eliminate artifacts caused by variations in sample pellet thickness and concentration during ex-situ measurements, all raw spectra were normalized using the invariant aromatic C–H deformation band of the MIL-101 framework at ~1018 cm^{-1} as an internal standard[S1]. Difference spectra were calculated by subtracting the normalized spectrum of the reference sample (e.g., MIL-101 or TAEA@MIL-101) from that of the target sample (e.g., TAEA@MIL-101 or TAEA@MIL-101-CO_2) to isolate the vibrational features of the grafted amines and chemisorbed species.

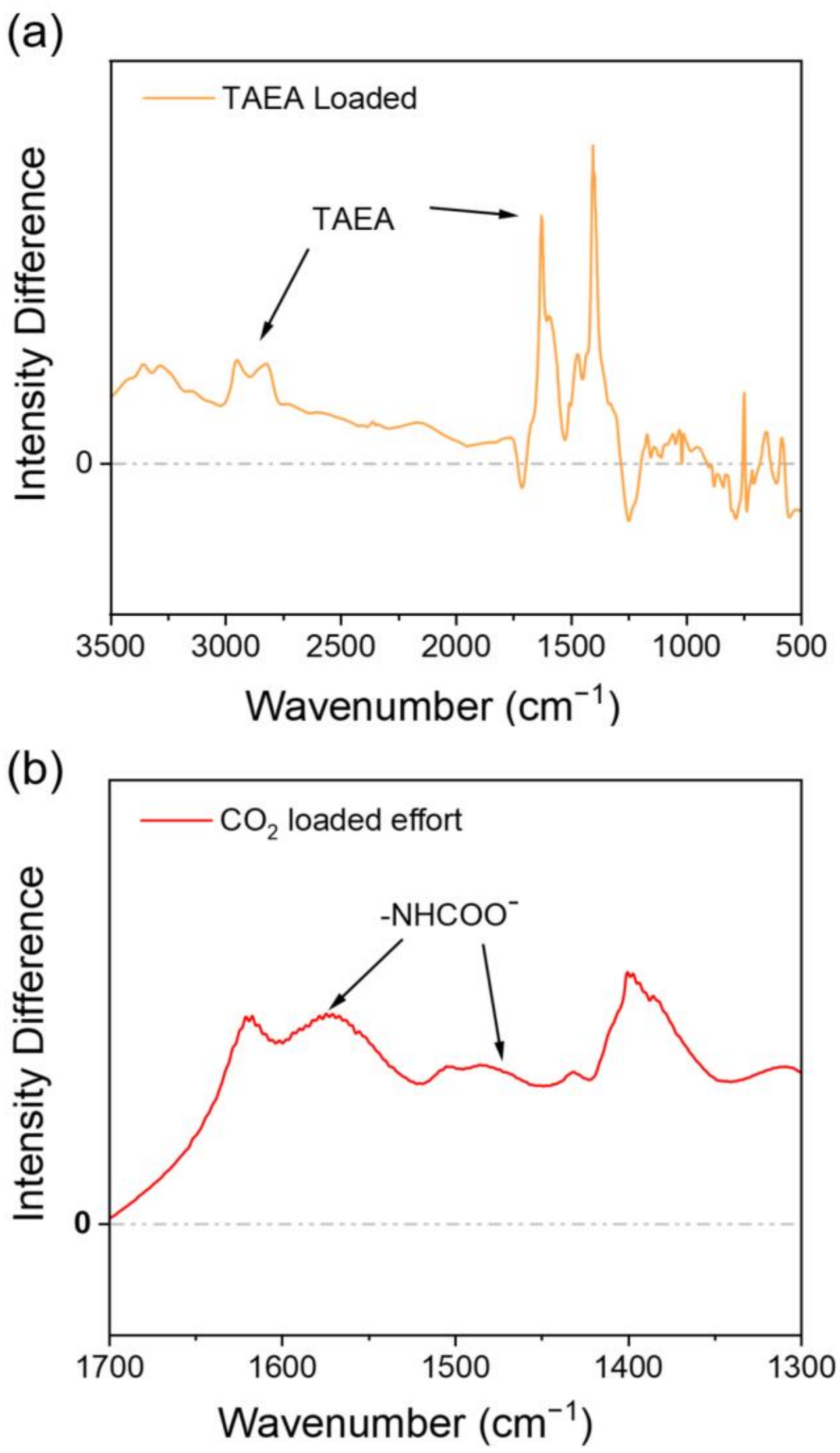

Figure S5. Normalized FTIR difference spectra calculated based on the internal standard band at 1018 cm^{-1}. (a) Effect of Amine load: Effect of Amine Grafting: Difference spectrum obtained by subtracting the normalized spectrum of MIL-101 from that of TAEA@MIL-101. The positive bands at 2950 cm^{-1} and ~3350 cm^{-1} correspond to the C–H stretching of the ethyl backbone and N–H stretching of the amine groups, respectively. (b) Effect of CO_2 Adsorption: Difference spectrum obtained by subtracting the normalized spectrum of TAEA@MIL-101 from that of CO_2-loaded TAEA@MIL-101. The distinct new peaks at 1595 cm^{-1} ($\nu_{as}COO^-$) and 1486 cm^{-1} ($\nu_{s}COO^-$) confirm the formation of alkylammonium carbamate species via chemisorption[S2].

Section S2. Dynamic Vapor Sorption (DVS) Measurements

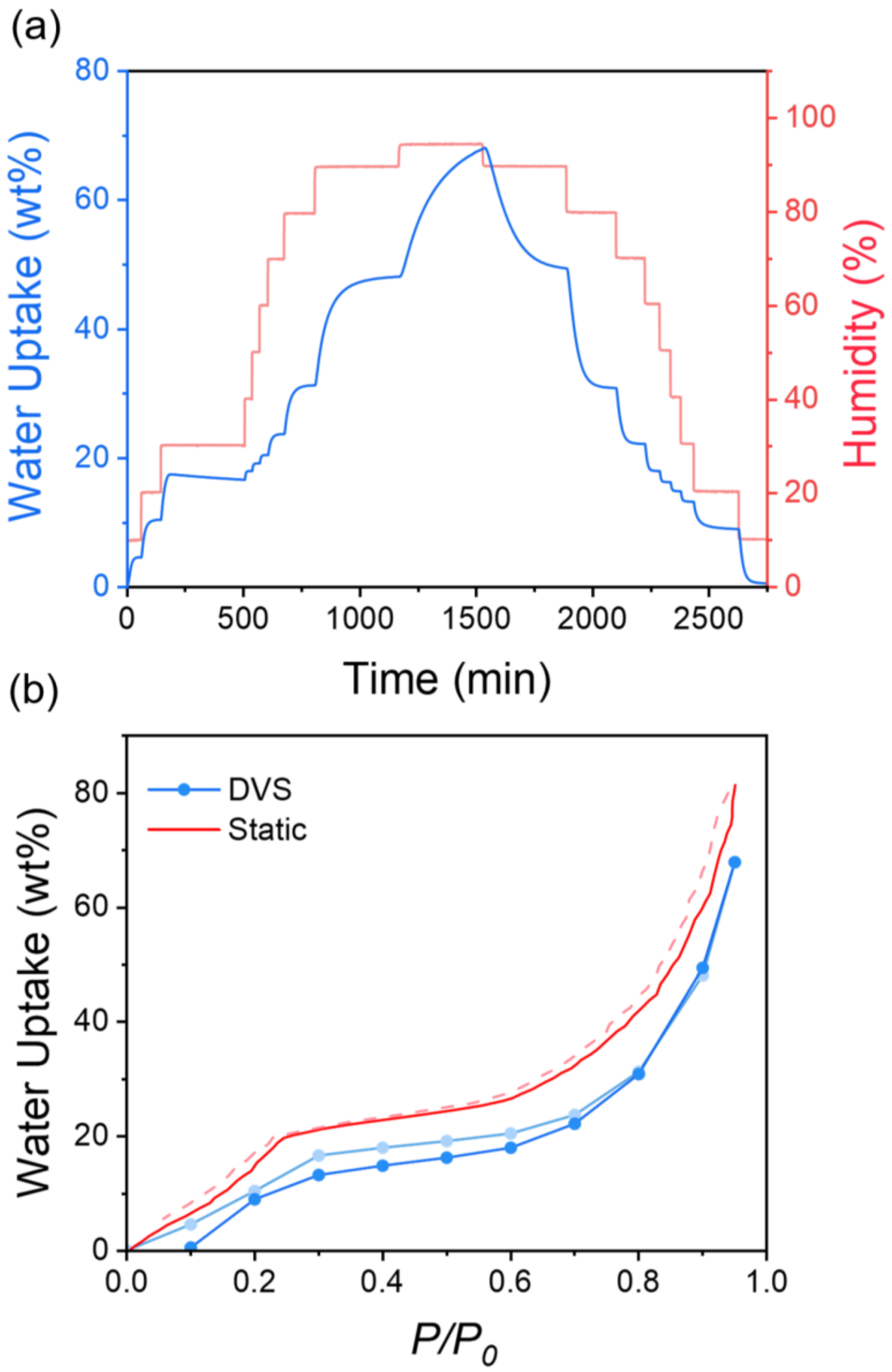

Figure S6. Dynamic water vapor sorption isotherms of TAEA@MIL-101 measured at 298 K.

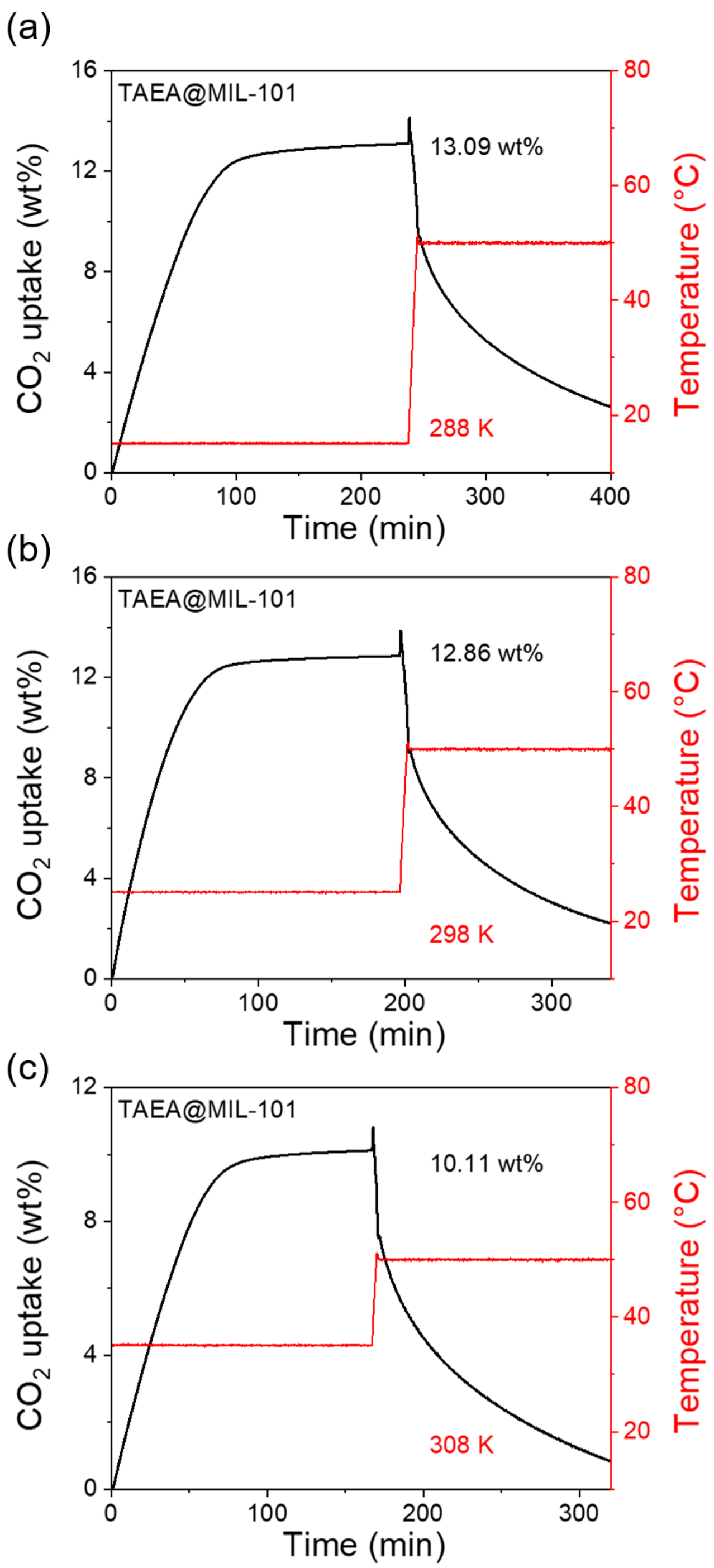

Figure S7. CO_2 uptake at 400 ppm at (a) 288 K, (b) 298 K, and (c) 308 K, showing rapid adsorption kinetics, followed by thermal regeneration. Teal curves (Left Axis): Represent the CO_2 uptake (wt%). The saturated capacities are marked as 13.09 wt% 12.86 wt% and 10.11 wt% respectively. Orange curves (Right Axis): Represent the temperature profile (°C).

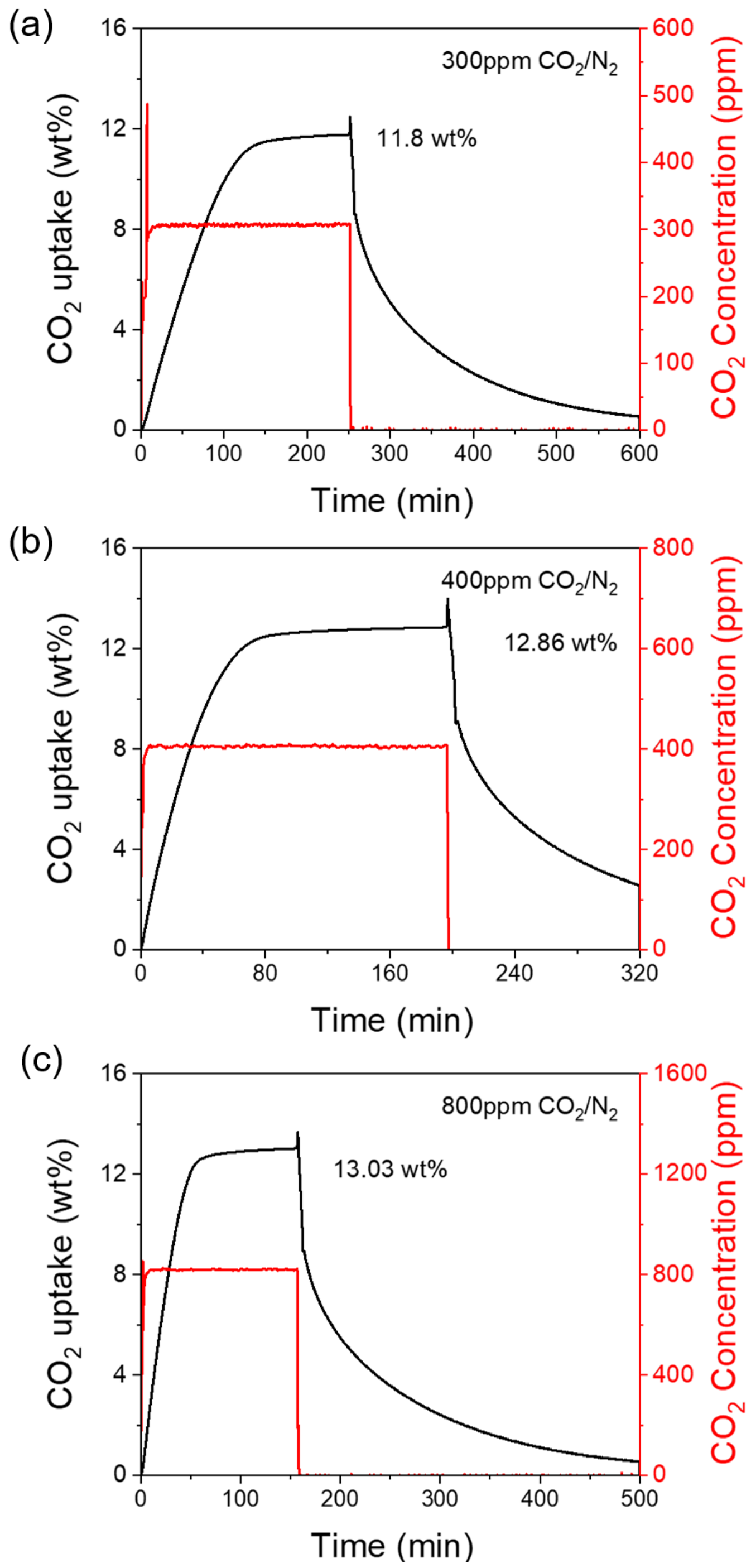

Figure S8. Dynamic adsorption and desorption profiles under different CO_2 concentrations, (a) 300 ppm CO_2/N_2, (b) 400 ppm CO_2/N_2 and (c) 800 ppm CO_2/N_2, confirming its applicability under different CO_2 concentrations conditions.

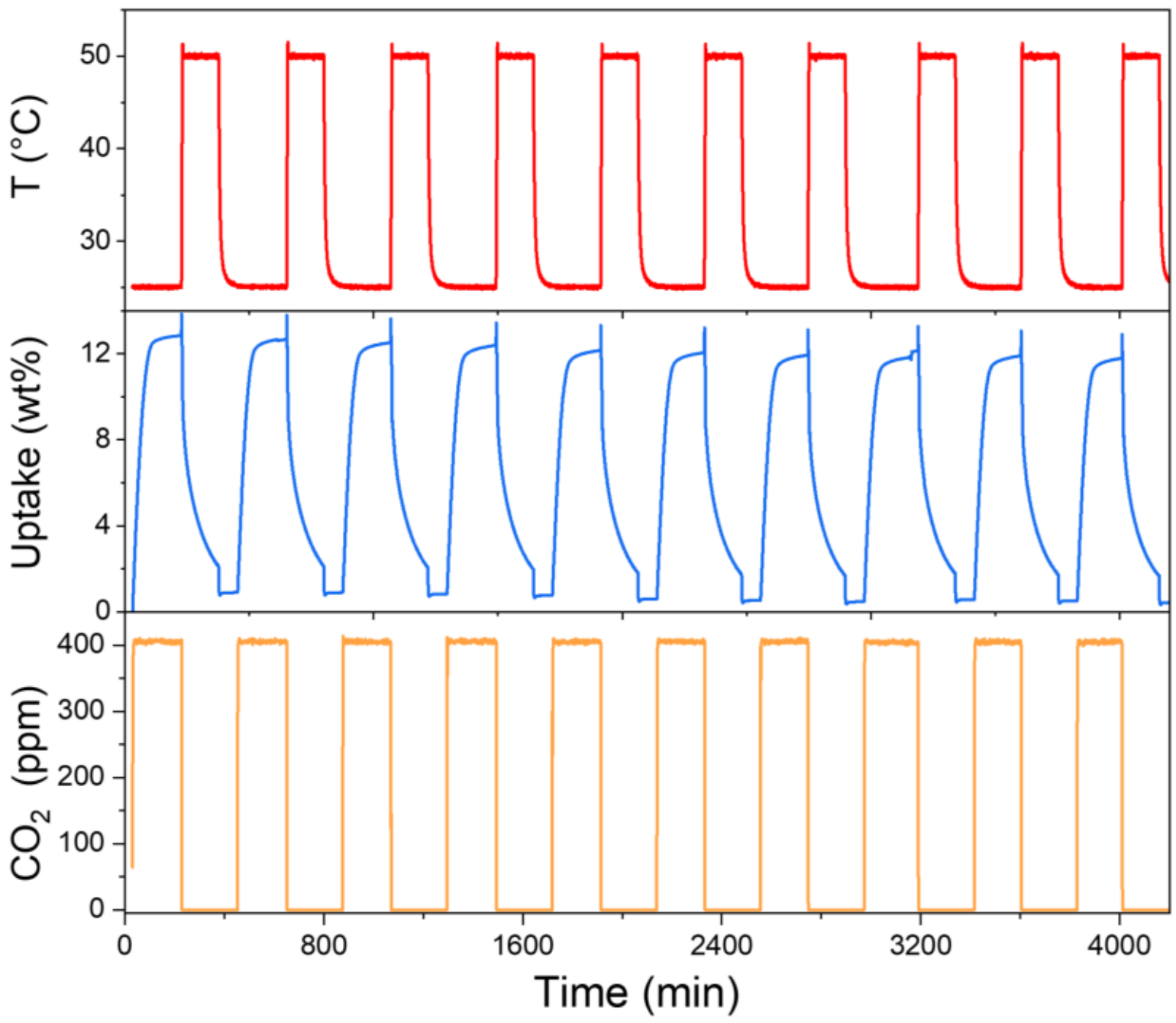

Figure S9. Cyclic adsorption-desorption profiles of TAEA@MIL-101under simulated air conditions (400 ppm CO_2). The panels display the temperature profile (top), CO_2 uptake capacity in wt% (middle), and CO_2 concentration profile (bottom) as a function of time, showing stable performance and low-temperature regeneration.

Section S3. CO_2/H_2O Competitive Adsorption Kinetics

To strictly evaluate and compare the adsorption rates, the kinetic data were analyzed based on the intraparticle diffusion model. According to the short-time approximation of Fick's second law for spherical particles, the fractional uptake (Mt/Me) during the initial adsorption stage is proportional to the square root of time, described by the following equation:

$$\frac{M_t}{M_e} = k \cdot t^{1/2} + C$$

Where:

Mt is the adsorption uptake as the time-dependent;

Me is the adsorption uptake at equilibrium;

k is the intraparticle diffusion rate constant (slope), representing the diffusion rate;

C is a constant related to the boundary layer effect.

We plotted the fractional uptake (Mt/Me) against $t^{1/2}$. To ensure the validity of the diffusion-controlled mechanism, the rate constant k (unit: $s^{1/2}$) was calculated from the slope of the range of Mt/Me < 0.6. The slope of this curve serves as the kinetic parameter, and the square of the ratio of the slopes for the two guest molecules constitutes the kinetic selectivity[S3].

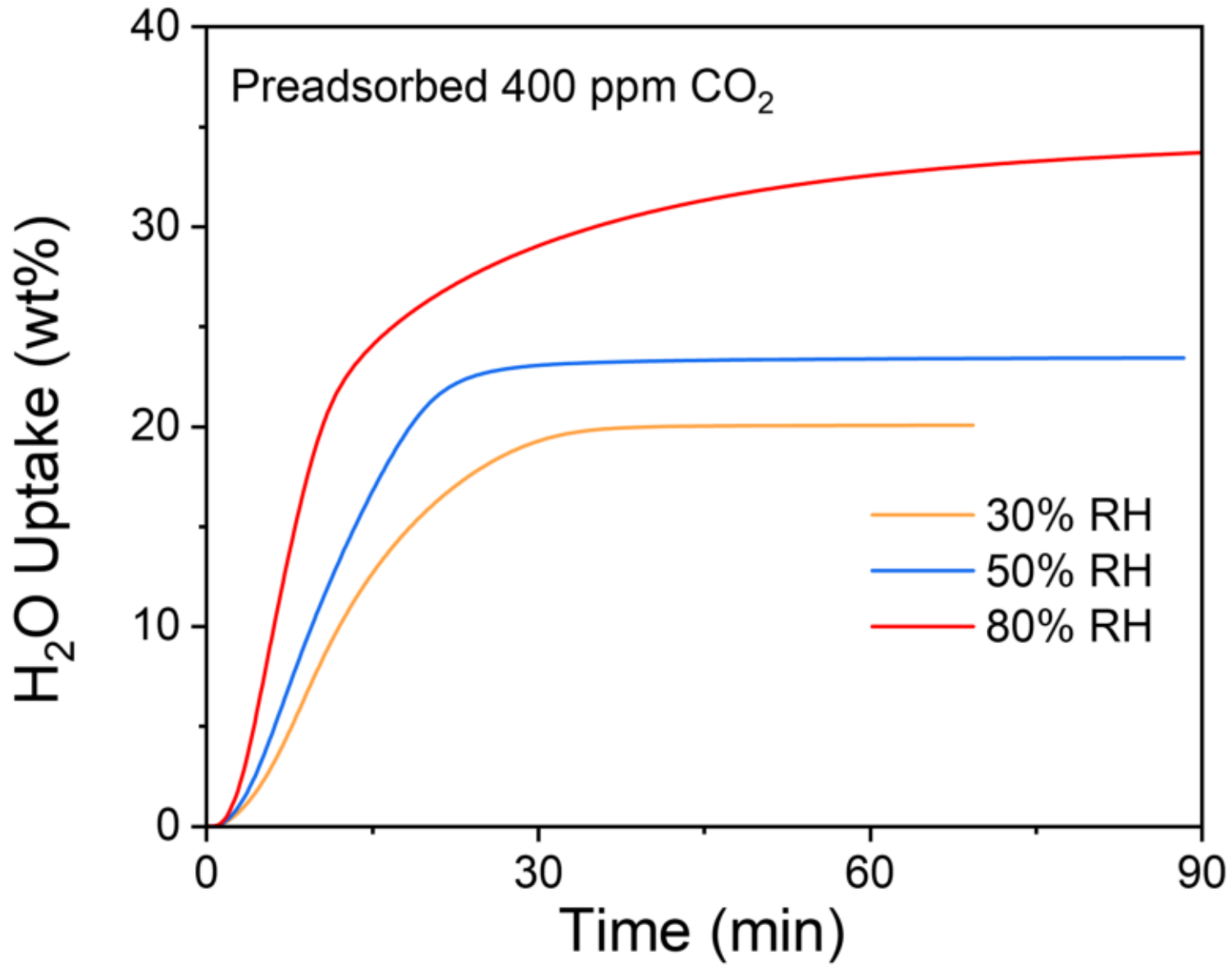

Figure S10. Water vapor adsorption profiles of TAEA@MIL-101 at different humidity levels. (30% RH, 50% RH, and 80% RH) after the material was preadsorbed 400 ppm CO_2, showing a significant increase in equilibrium capacity with rising humidity, exhibiting fast adsorption kinetics in the initial stage.

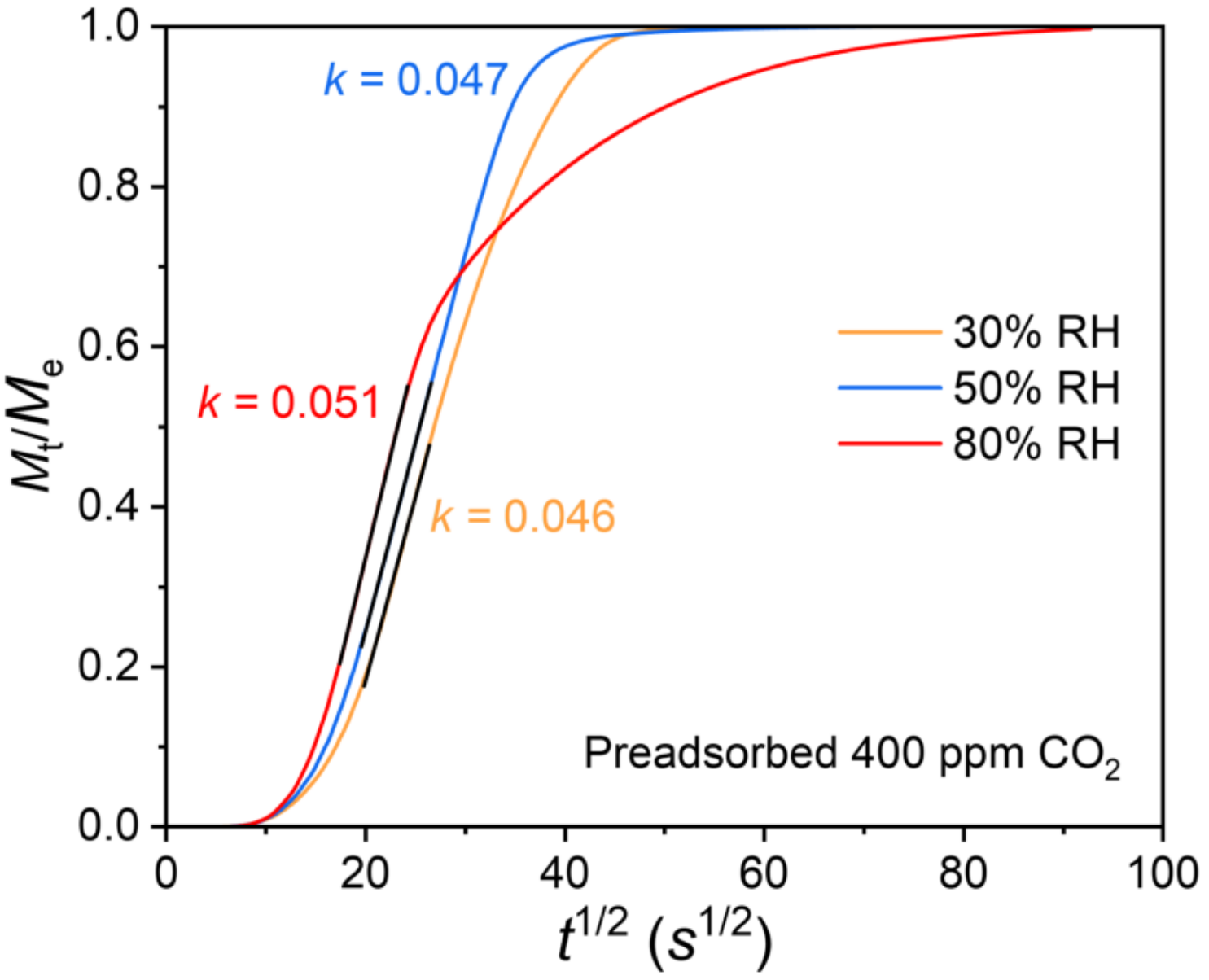

Figure S11. Kinetic analysis of water vapor adsorption on TAEA@MIL-101.

Table S2. The Slope Obtained from the Kinetic Curves by Linear Fitting and The CO_2/H_2O Kinetic Curves of The Materials.

Adsorption Conditions	wt%	slope	Kinetic Selectivity
300 ppm CO_2	11.8	0.01580	/
400 ppm CO_2	12.86	0.02061	/
800 ppm CO_2	13.03	0.02435	/
30% RH	22.21	0.02701	1.72
50% RH	25.81	0.05519	7.17
80% RH	38.33	0.06063	8.65

Table S3. Kinetic Parameters for CO_2 Re-Adsorption on TAEA@MIL-101 after Exposure to Different Humidity Levels. The samples were pre-exposed to the specified RH conditions, followed by desorption, and then subjected to CO_2 adsorption tests (400 ppm). The kinetic parameters reflect the material's performance retention after humidity exposure.

Adsorption Conditions	wt%	slope	R^2
400 ppm CO_2	12.86	0.02061	0.999
400ppm CO_2-preadsorbed 30% RH	8.95	0.01577	0.999
400ppm CO_2-preadsorbed 50% RH	8.21	0.01963	0.999
400ppm CO_2-preadsorbed 80% RH	6.14	0.02167	0.999

Table S4. Kinetic Parameters for Water Vapor Adsorption on CO_2-Pre-Saturated TAEA@MIL-101. The samples were first saturated with CO_2 (400 ppm), followed by the introduction of humid streams at different RH levels. This table evaluates the kinetics of water co-adsorption or displacement on CO_2-loaded samples.

**Adsorption Conditions**	**wt%**	**slope**	**R^2**
30%RH-preadsorbed 400 ppm CO_2	20.05	0.04587	0.999
50%RH-preadsorbed 400 ppm CO_2	22.43	0.047	0.999
80%RH-preadsorbed 400 ppm CO_2	34.41	0.05095	0.999

Section S4. Mimic Direct Air Capture Environment Adsorption Breakthrough Measurements

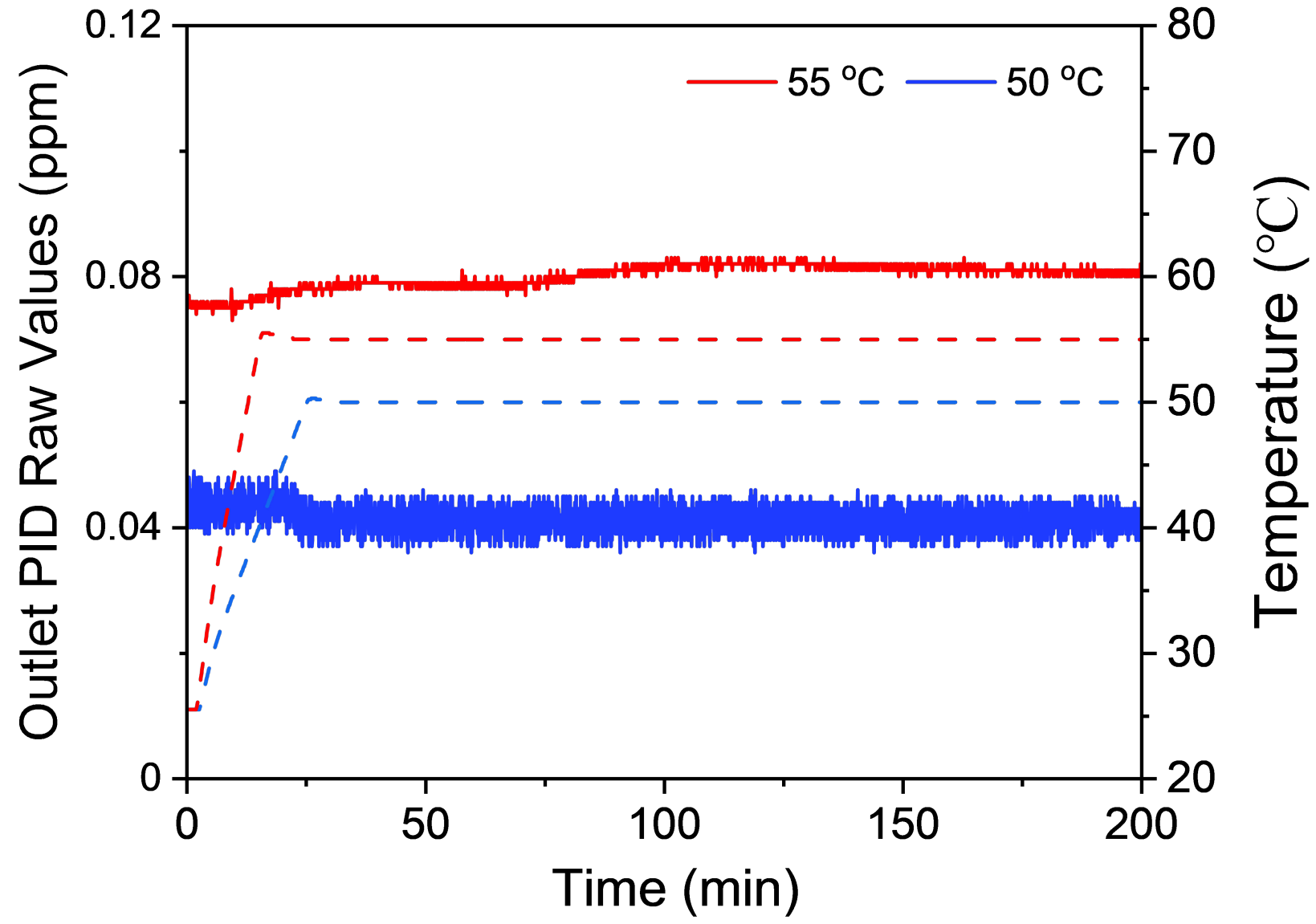

Figure S12. Monitoring of amine thermal stability for TAEA-loaded MIL-101 during the CO_2 desorption process.

Section S5. Comparative Analysis of CO_2 Capture Performance

Table S5. Summary of DAC CO_2 uptake capture performances of various adsorbents. (corresponding to the comparison in Figure 4g).

Type	Name	Capacity (mmol/g) 298K 400 ppm CO_2	Ref.
Zeolites	Ca-LTA-3	1.2	S4
	Na-Gis(1.5Lys)	0.67	S5
	Fe@13X	0.64	S6
	Mn-LTA-3	0.54	S7
	Na-CHA(1.5Lys)	0.46	S5
	13X	0.29	S6
Amine-inorganic	LDH-MMO-M/45PM	1.82	S8
	PEI5O/MMO-M	1.52	S8
	ED-PEI/PEG@FS-TBP	1.49	S9
	FS-PEI-33%	1.18	S10
	MMO-M/45PM	0.43	S8
physisorptive MOFs	NbOFFIVE-1-Ni	1.3	S11
	SIFSIX-3-Cu	1.24	S12
	TIFSIX-3-Ni	1.11	S13
	TIFSIX-3-Co	1.05	S14
	SIFSIX-3-Ni	0.42	S15
	SIFSIX-3-Zn	0.13	S15
Amine MOFs	TAEA@MIL-101	3.12	This work
	$[Mg_2(hob)(mmen)_{1.88}(H_2O)_{0.12}]$	2.91	S16
	$[Mg_2(dobpdc)(en)_{1.6}]$	2.83	S17
	$[Mg_2(hob)(nmen)_{1.78}(H_2O)_{0.22}]$	2.6	S16
	$[Mg_2(hob)(een)_{1.95}(H_2O)_{0.05}]$	2.49	S16
	TEPA-MIL-101(Cr)-50%	2.14	S18
	$[Mg_2(dobpdc)(mmen)_{1.6}]$	2	S19
	$[Mg_2(dobpdc)(pn)_{1.7}(H_2O)_{0.3}]$	1.76	S20
	$[Mg_2(dobpdc)(epn)_{1.9}(H_2O)_{0.1}]$	1.76	S20
	$[Mg_2(dobpdc)(nmpn)_{1.9}(H_2O)_{0.1}]$	1.76	S20
	PEI-MIL-101(Cr)	1.68	S18

Mg_2 (dobdc)(en)0.33	1.51	S21
MIL-101(Cr)-MEA-100	1.5	S22
[Mg_2 (dobpdc)(nmen)$_{1.7}$(H_2 O)$_{1.5}$]	0.76	S23
MOF-808-TAPA	0.498	S24
[Mg_2(dobpdc)(men)$_{1.6}$]	0.41	S25
MOF-808-TAEA	0.348	S24
MOF-808-Arg	0.342	S24
MOF-808-EDA	0.271	S24
MOF-808-Pro	0.175	S24
MOF-808-DAP	0.175	S24
MOF-808-Lys	0.162	S24
MOF-808-Gly	0.142	S24

Table S6. Comparison of Comprehensive Performance Parameters between TAEA@MIL-101(Cr) and Other Relevant Adsorbents (Corresponding to the Radar Chart in Figure 4f).

Factors	TAEA@MIL-101	TEPA-MIL-101(Cr)-50%	mmen-Mg_2(hob)
K ($s^{-1/2}$)	**0.0206**	0.01316	0.00691
Adsorption Capacity (mmol/g)	**2.92**	2.19	2.91
Regeneration Temperature (°C)	**50**	60	70
Amine Efficiency (mol CO_2/mol N)	**0.21**	0.16	0.32
Stability	**10 cycles ~92%**	over 5 cycles~95%[a]	10 cycles ~100%
Ref.	**this work**	S18	S16

Data for reference materials are cited from the corresponding literature.

Specifically, to ensure a consistent comparison in Figure 5e, the kinetic rate constants (k) for all reference materials were calculated in this work based on their reported uptake profiles, following the intraparticle diffusion model described in Section S4.

[a] The cyclic stability data for TEPA-MIL-101(Cr)-50% (>95% retention over 5 cycles) was obtained under sub-ambient adsorption conditions at −20 °C and regeneration at 60 °C.

Amine efficiency is defined as the molar ratio of the CO_2, adsorption capacity to amines (in terms of N), and the calculation equation is mentioned as follows

$$n = \frac{q}{m_N}$$

where q is the CO_2 adsorption capacity, mmol/g, and m_N is the amount of amine (in terms of N) in the adsorbent, mmol/g[26].

Table S7. Reproducibility of CO_2 Adsorption Capacity for TAEA@MIL-101

Batch No.	400 ppm CO_2 Capacity (mmol/g)
1	3.38
2	3.25
3	3.17
4	3.12
5	3.07
6	3.06
7	3.04
8	3.03
9	3.03
10	3.02

References

S1. Férey, G.; Mellot-Draznieks, C.; Serre, C.; Millange, F.; Dutour, J.; Surblé, S.; Margiolaki, I. A chromium terephthalate-based solid with unusually large pore volumes and surface area. *Science* **2005**, *309*, 2040–2042.

S2. Bacsik, Z.; Ahlsten, N.; Ziadi, A.; Zhao, G.; Garcia-Bennett, A. E.; Martín-Matute, B.; Hedin, N. Mechanisms and Kinetics for Sorption of CO_2 on Bicontinuous Mesoporous Silica Modified with n-Propylamine. *Langmuir* **2011**, *27*, 11118–11128.

S3. Shi, Z.; Tao, Y.; Wu, J.; Zhang, C.; He, H.; Long, L.; Lee, Y.; Li, T.; Zhang, Y.-B. Robust Metal–Triazolate Frameworks for CO_2 Capture from Flue Gas. *J. Am. Chem. Soc.* **2020**, *142*, 2750–2754.

S4. Tao, Z.; Tian, Y.; Ou, S. Y.; Gu, Q.; Shang, J. Direct air capture of CO_2 by metal cation‐exchanged LTA zeolites: Effect of the charge-to-size ratio of cations. *AlChE J.* **2023**, *69*: e18139.

S5. Kencana, K. S.; Kemp, K. C.; Hong, S. B. L-lysine-assisted synthesis of gismondine and chabazite nanozeolites for direct air capture of CO_2. *Sep. Purif. Technol.* **2024**, *329*.

S6. Xiang, X.; Guo, T.; Yin, Y.; Gao, Z.; Wang, Y.; Wang, R.; An, M.; Guo, Q.; Hu, X. High Adsorption Capacity Fe@13X Zeolite for Direct Air CO_2 Capture. *Ind. Eng. Chem. Res.* **2023**, *62*, 5420–5429.

S7. Tian, C.; Liu, X.; Liu, C.; Li, S.; Li, Q.; Sun, N.; Gao, K.; Jiang, Z.; Chang, K.; Xuan, Y. Air to fuel: Direct capture of CO_2 from air and in-situ solar-driven conversion into syngas via Ni_x/NaA nanomaterials. *Nano Res.* **2023**, *16*, 10899–10912.

S8. Shao, Q.; Gan, Z.; Ge, B.; Liu, X.; Chen, C.; O'Hare, D.; Zhu, X. 3D printing of poly(ethyleneimine)-functionalized Mg-Al mixed metal oxide monoliths for direct air capture of CO_2. *J. Energy Chem.* **2024**, *96*, 491–500.

S9. Huang, J.; Huang, Y.; Yu, X.; Zhao, Y.; Wu, X.; Liang, P.; Li, Z.; Wang, H.; Xia, Q. Advanced Solid Amine Adsorbents with Exceptional Oxidative Stability for Efficient Capture of Low‐concentration CO_2. *Small* **2024**, *21*: 2411010.

S10. Goeppert, A.; Czaun, M.; May, R. B.; Prakash, G. K. S.; Olah, G. A.; Narayanan, S. R. Carbon Dioxide Capture from the Air Using a Polyamine Based Regenerable Solid Adsorbent. *J. Am. Chem. Soc.* **2011**, *133*, 20164–20167.

S11. Kumar, A.; Hua, C.; Madden, D. G.; O'Nolan, D.; Chen, K.-J.; Keane, L.-A. J.; Perry, J. J.; Zaworotko, M. J. Hybrid ultramicroporous materials (HUMs) with enhanced stability and trace carbon capture performance. *Chem. Commun.* **2017**, *53*, 5946–5949.

S12. Shekhah, O.; Belmabkhout, Y.; Chen, Z.; Guillerm, V.; Cairns, A.; Adil, K.; Eddaoudi, M. Made-to-order metal-organic frameworks for trace carbon dioxide removal and air capture. *Nat. Commun.* **2014**, *5*: 4228.

S13. Low, M.-Y. A.; Danaci, D.; Azzan, H.; Jiayi, A. L.; Yong, G. W. S.; Itskou, I.; Petit, C. Physicochemical Properties, Equilibrium Adsorption Performance, Manufacturability, and Stability of TIFSIX-3-Ni for Direct Air Capture of CO_2. *Energy Fuels* **2024**, *38*, 11947–11965.

S14. Zhang, Z.; Ding, Q.; Cui, J.; Cui, X.; Xing, H. High and selective capture of low-concentration CO_2 with an anion-functionalized ultramicroporous metal-organic framework. *Sci. China Mater.* **2020**, *64*, 691–697.

S15. Nugent, P.; Belmabkhout, Y.; Burd, S. D.; Cairns, A. J.; Luebke, R.; Forrest, K.; Pham, T.; Ma, S.; Space, B.; Wojtas, L.; Eddaoudi, M.; Zaworotko, M. J. Porous materials with optimal adsorption thermodynamics and kinetics for CO_2 separation. *Nature* **2013**, *495*, 80–84.

S16. Choe, J. H.; Kim, H.; Yun, H.; Kurisingal, J. F.; Kim, N.; Lee, D.; Lee, Y. H.; Hong, C. S. Extended MOF-74-Type Variant with an Azine Linkage: Efficient Direct Air Capture and One-Pot Synthesis. *J. Am. Chem. Soc.* **2024**, *146*, 19337–19349.

S17. Lee, W. R.; Hwang, S. Y.; Ryu, D. W.; Lim, K. S.; Han, S. S.; Moon, D.; Choi, J.; Hong, C. S. Diamine-functionalized metal–organic framework: exceptionally high CO_2 capacities from ambient air and flue gas, ultrafast CO_2 uptake rate, and adsorption mechanism. *Energy Environ. Sci.* **2014**, *7* 744–751.

S18. Rim, G.; Kong, F.; Song, M.; Rosu, C.; Priyadarshini, P.; Lively, R. P.; Jones, C. W. Sub-Ambient Temperature Direct Air Capture of CO_2 using Amine-Impregnated MIL-101(Cr) Enables Ambient Temperature CO_2 Recovery. *JACS Au* **2022**, *2*, 380–393.

S19. McDonald, T. M.; Mason, J. A.; Kong, X.; Bloch, E. D.; Gygi, D.; Dani, A.; Crocellà, V.; Giordanino, F.; Odoh, S. O.; Drisdell, W. S.; Vlaisavljevich, B.; Dzubak, A. L.; Poloni, R.; Schnell, S. K.; Planas, N.; Lee, K.; Pascal, T.; Wan, L. F.; Prendergast, D.; Neaton, J. B.; Smit, B.; Kortright, J. B.; Gagliardi,

L.; Bordiga, S.; Reimer, J. A.; Long, J. R. Cooperative insertion of CO_2 in diamine-appended metal-organic frameworks. *Nature* **2015**, *519*, 303–308.
S20. Park, J.; Park, J. R.; Choe, J. H.; Kim, S.; Kang, M.; Kang, D. W.; Kim, J. Y.; Jeong, Y. W.; Hong, C. S. Metal–Organic Framework Adsorbent for Practical Capture of Trace Carbon Dioxide. *ACS Appl. Mater. Interfaces* **2020**, *12*, 50534–50540.
S21. Choi, S.; Watanabe, T.; Bae, T.-H.; Sholl, D. S.; Jones, C. W. Modification of the Mg/DOBDC MOF with Amines to Enhance CO_2 Adsorption from Ultradilute Gases. *J. Phys. Chem. Lett.* **2012**, *3*, 1136–1141.
S22. Jiang, K.; Yang, J.; Zhou, Y.; Gu, J. Synergetic enhancement of CO_2 direct air capture with monoethanolamine-impregnated MIL-101(Cr) MOFs. *Microporous Mesoporous Mater.* **2024**, *366*: 112920.
S23. Lee, W. R.; Kim, J. E.; Lee, S. J.; Kang, M.; Kang, D. W.; Lee, H. Y.; Hiremath, V.; Seo, J. G.; Jin, H.; Moon, D.; Cho, M.; Jung, Y.; Hong, C. S. Diamine - Functionalization of a Metal - Organic Framework Adsorbent for Superb Carbon Dioxide Adsorption and Desorption Properties. *ChemSusChem* **2018**, *11*, 1694–1707.
S24. Chen, O. I.-F.; Liu, C.-H.; Wang, K.; Borrego-Marin, E.; Li, H.; Alawadhi, A. H.; Navarro, J. A. R.; Yaghi, O. M. Water-Enhanced Direct Air Capture of Carbon Dioxide in Metal–Organic Frameworks. *J. Am. Chem. Soc.* **2024**, *146*, 2835–2844.
S25. Jo, H.; Lee, W. R.; Kim, N. W.; Jung, H.; Lim, K. S.; Kim, J. E.; Kang, D. W.; Lee, H.; Hiremath, V.; Seo, J. G.; Jin, H.; Moon, D.; Han, S. S.; Hong, C. S. Fine-Tuning of the Carbon Dioxide Capture Capability of Diamine-Grafted Metal–Organic Framework Adsorbents Through Amine Functionalization. *ChemSusChem* **2017**, *10*, 541–550.
S26. Chen, X.; Leng, J.; Ma, F.; Wu, J.; Jin, Y.; He, F.; Yang, C.; Huang, H.; Liu, X.; Duan, C.; Shang, S.; Ye, D. Constructing Amine-Functionalized Hierarchically Porous Porphyrin-Based Metal–Organic Frameworks for Highly Enhanced Direct Air Capture of CO_2. *ACS Appl. Mater. Interfaces* **2025**, *17*, 48360–48374.
